# Ortho­rhom­bic cerium(III) carbonate hydroxide studied by synchrotron powder X-ray diffraction

**DOI:** 10.1107/S2056989025003512

**Published:** 2025-05-15

**Authors:** Liam A. V. Nagle-Cocco, Robert T. Bell, Nicholas A. Strange

**Affiliations:** a Stanford Synchrotron Radiation Lightsource SLAC National Accelerator, Laboratory,Menlo Park California 94025 USA; b National Renewable Energy Laboratory, Golden, Colorado, 80401, USA; Vienna University of Technology, Austria

**Keywords:** crystal structure, synchrotron radiation, carbonate, precursor, powder diffraction, isotypism

## Abstract

Cerium(III) carbonate is a precursor material for the synthesis of various Ce-containing compounds. In this work, a synchrotron powder X-ray diffraction study of commercially obtained ‘cerium(III) carbonate hydrate’ indicates that multiple Ce-containing phases are present. The majority phase CeCO_3_OH (52.49%_wt_) was refined using an ortho­rhom­bic *Pmcn* structure model with *a* = 5.01019 (2) Å, *b* = 8.55011 (4) Å and *c* = 7.31940 (4) Å, which is based on a reported structure for the lanthanoid carbonate mineral ancylite. Additionally, a substantial portion of the precursor material is cubic cerium(IV) oxide (47.12%_wt_).

## Abstract 2

A synchrotron powder X-ray diffraction study of commercially obtained ‘cerium(III) carbonate hydrate’ indicates that multiple Ce-containing phases coexist, none of which are Ce_2_(CO_3_)_3_. The majority phase is an ortho­rhom­bic phase of composition CeCO_3_OH.

## 1. Chemical context

Cerium(III) carbonate is a precursor material in the synthesis of various materials, such as the (Ca,Ce)(Ti,Mn)O_3_ (Wexler *et al.*, 2023 ▸) and Ba_4_CeMn_3_O_12_ (Bell *et al.*, 2022 ▸) perovskites, which are of inter­est for solar thermochemical hydrogen (STCH) production. There are no reported crystal structures deposited in the Inorganic Crystal Structure Database (ICSD; Zagorac *et al.* 2019 ▸) with the nominal Ce_2_(CO_3_)_3_ composition; instead, reported structures typically include H_2_O or hydroxide groups. For instance, a hexa­gonal cerium(III) carbonate hydroxide, CeCO_3_OH, has been reported from a mineralogical study (ICSD 238537; Michiba *et al.*, 2013 ▸), and an ortho­rhom­bic hydrated carbonate phase, Ce_2_(CO_3_)_3.8_H_2_O, has been synthesized (ICSD 410859; Daiguebonne *et al.*, 2001 ▸). A precise accounting for the hydroxide and hydrate groups in precursor compounds is necessary for the synthesis, as the calculation of correct mass ratios is dependent on accurate knowledge of the mass per formula unit.

**Figure 1:**
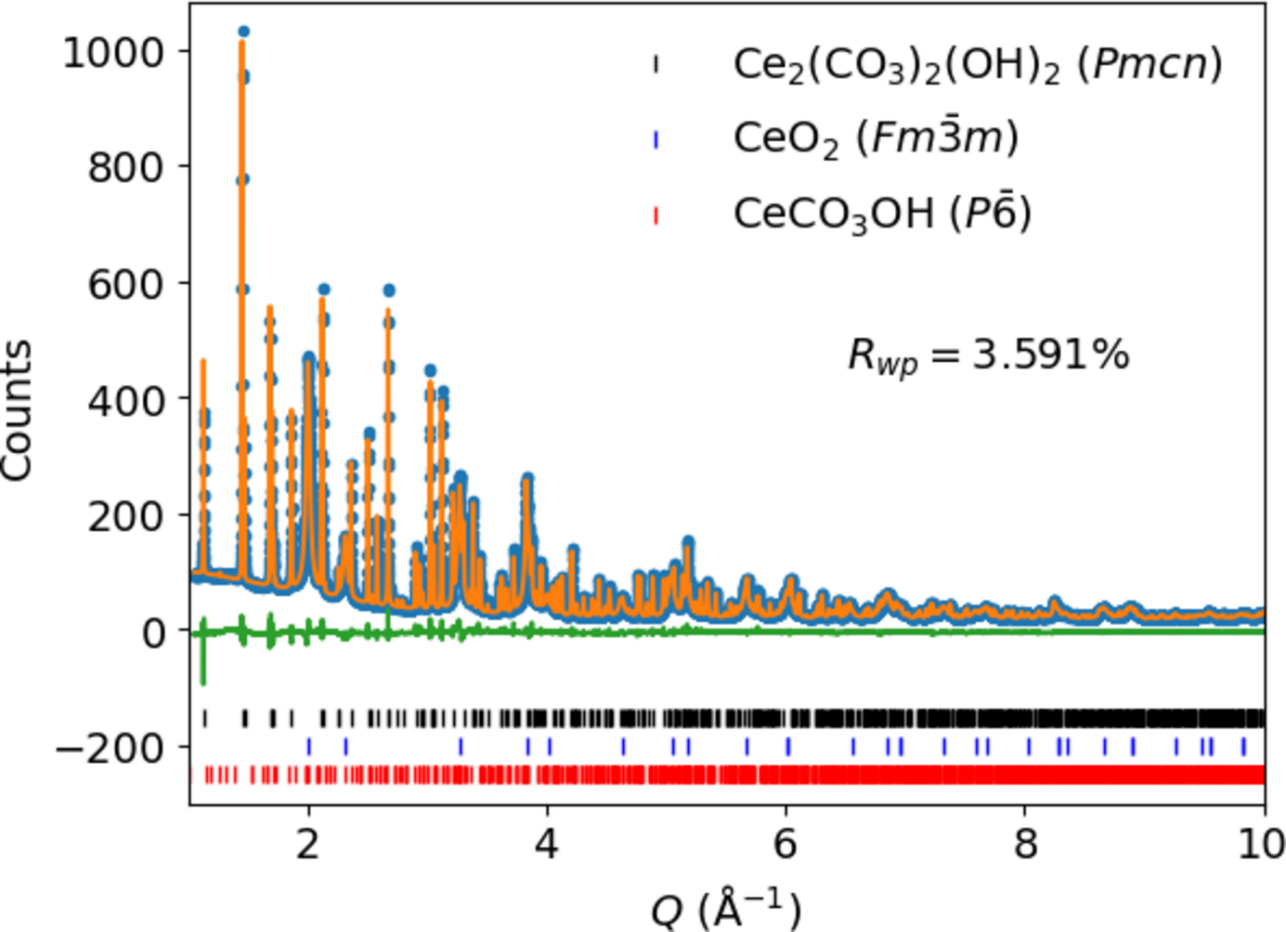
Synchrotron powder X-ray diffraction data, as a function of momentum transfer *Q*, for the studied ‘cerium(III) carbonate hydrate’ sample (blue points), over which is superimposed a triphasic calculated diffraction pattern (orange) consisting of the Rietveld-refined ortho­rhom­bic *Pmcn* CeCO_3_OH phase (52.49%_wt_), cubic *Fm*
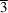
*m* CeO_2_ (47.12%_wt_), and previously reported hexa­gonal *P*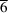 CeCO_3_OH (0.40%_wt_). The difference pattern is shown in green.

In this work, part of a project investigating Ce-containing perovskites for STCH production, we sought to characterize precisely the different crystalline phases present in a commercially obtained ‘cerium(III) carbonate hydrate’ precursor material. The synchrotron X-ray diffraction pattern collected on the ‘cerium(III) carbonate hydrate’ sample is shown in Fig. 1 ▸. We found that the experimental diffraction pattern was complex, and could not be fit solely using structures deposited in the ICSD. We also found that the sample, in fact, does not correspond to ‘cerium(III) carbonate hydrate’ but is a mixture of three other phases.

Many of the Bragg reflections could be attributed to a cerium(IV) oxide phase with cubic symmetry in space group *Fm*
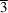
*m* (ICSD 24887; Ma *et al.*, 2020 ▸). Some low-intensity reflections could be attributed to the previously reported phase CeCO_3_OH with hexa­gonal *P*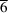 symmetry (ICSD 238537; Michiba *et al.*, 2013 ▸). The remaining prominent reflections could not be fitted with any phase reported in the ICSD. An ortho­rhom­bic cerium carbonate hydroxide phase based on the *Ln*CO_3_OH structural template of the mineral ancylite (ICSD 4242; Dal Negro *et al.*, 1975 ▸) was used as a starting point to fit these reflections with space group *Pmcn*. After independently solving the *Pmcn* structure, we found that a *Pmcn* cerium carbonate hydroxide structure has been reported previously, for instance by Wei *et al.* (2021 ▸). However, this structure was not deposited in the ICSD and atomic positions were not reported.

## 2. Structural commentary

**Figure 2:**
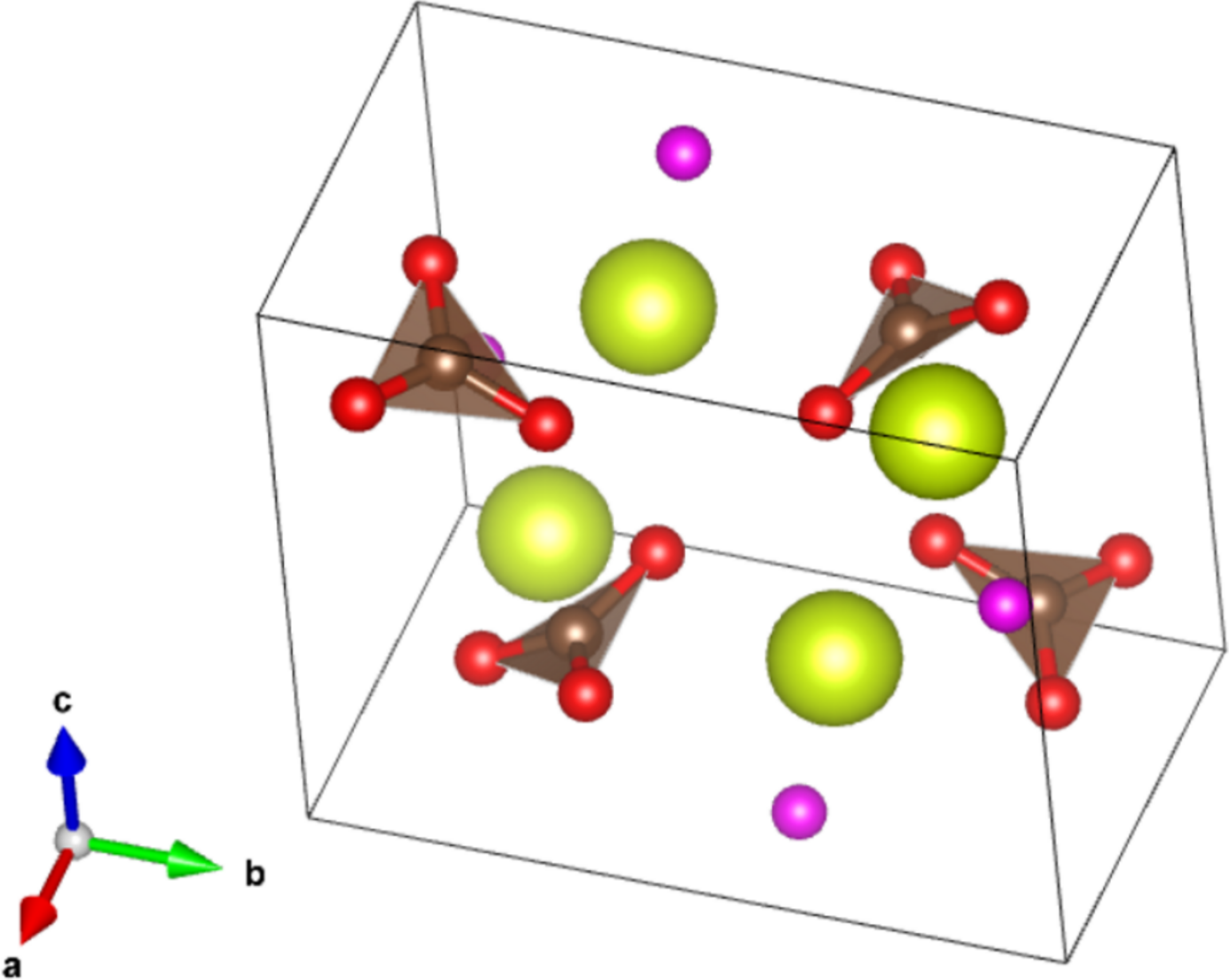
The unit cell of the ortho­rhom­bic cerium(III) carbonate hydroxide phase. Color code: Ce: yellow. CO_3_ triangles: brown planes with red vertices; O within hydroxide group: pink. Note that H atoms were not included in the refinement and so are not included in the structure.

The crystal structure of the newly-reported CeCO_3_OH phase is visualized in Fig. 2 ▸. The Ce1 atom, the C1 atom and two of the O atoms (O1, O3) are situated at a special position with point group symmetry *m* (multiplicity 4, Wyckoff letter *c*), and the third O atom (O2) is situated at a general position (8 *d*).

**Figure 3:**
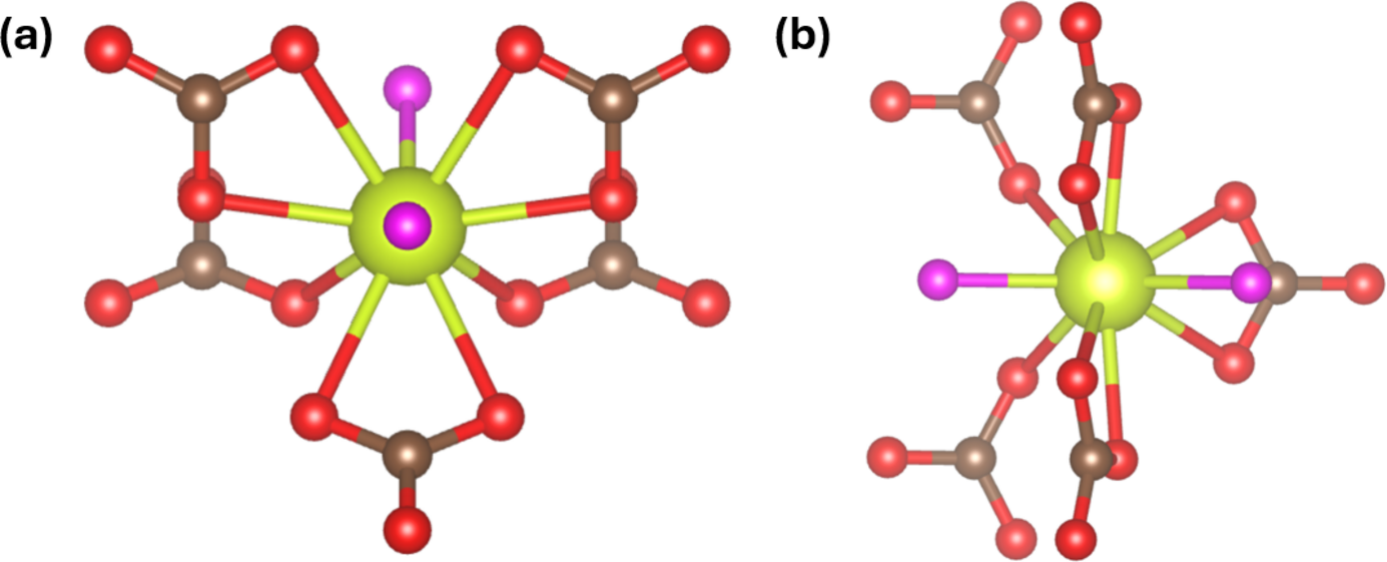
The coordination sphere of the Ce^III^ cations in the ortho­rhom­bic CeCO_3_OH phase from two different angles shown in (*a*) and (*b*). Color codes are as in Fig. 2 ▸.

The coordination sphere around Ce1 is shown in Fig. 3 ▸. Each Ce^III^ cation has five CO_3_ groups coordinating to it; with three of these, two O atoms are chelating the Ce^III^ cation with two O atoms [with Ce—O bond lengths of 2.5697 (8) Å and 2.723 (2) Å], and with the other two CO_3_ groups only a single O is bonded to the Ce^III^ cation [with Ce—O bond length of 2.650 (3) Å]. Additionally, each Ce^3+^ cation has two OH^−^ groups bonded to it *via* the O atom [with Ce—O bond lengths of 2.396 (3) Å and 2.458 (3) Å]. This indicates a coordination number of 10 and an average bond length of 2.606 Å, well within the mean bond length and its standard deviation of 2.595±0.098 Å reported by Gagné (2018 ▸) for CeO_10_ coord­in­ation polyhedra.

Within the carbonate CO_3_ group, the C—O bonds are between 1.262 (7) and 1.293 (4) Å, which is typical of reported C—O bond lengths for carbonates (mean 1.284 Å with standard deviation ±0.020 Å; Gagné & Hawthorne, 2018 ▸).

The H-atom position of the OH^−^ group was not located and the H atom is not included in the finally modeled structure. We therefore make no comment regarding O—H bond lengths or hydrogen-bonding inter­actions.

## 3. Database survey

The reported ortho­rhom­bic CeCO_3_OH is a synthetic analog to the naturally occurring mineral ancylite (ICSD 4242; Dal Negro *et al.*, 1975 ▸), in which the cation site is typically occupied by some combination of different lanthanoids and the hydroxide group may be partially substituted by a hydrate group (Belovitskaya *et al.*, 2002 ▸). In the mineral form, non-rare earths such as Ca (ICSD 94065; Petersen *et al.*, 2001 ▸) and Pb (ICSD 125071; Wu *et al.*, 2023 ▸) have also been reported on the Ce site.

Amongst the isotypic lanthanoid carbonate hydroxide phases (*Ln* = La, Pr, Nd, Sm, Eu, Gd) with a 1:1:1 ratio of metal:carbonate:hydroxide, the reported structure in this work is fairly typical, in that it is an ortho­rhom­bic cell with four *Ln* cations per unit cell, and has two OH groups bonded to each *Ln*, and 5 CO_3_ groups bonded to each *Ln* with a 3:2 ratio of CO_3_ groups with two and one O—*Ln* bonds respectively (Hämmer & Höppe, 2019 ▸).

The reported CeCO_3_OH structure differs from some other metal carbonate hydroxide phases, however. For instance, it is quite different to a reported zinc carbonate hydroxide phase (hydro­zincite with ideal formula Zn_5_(CO_3_)_2_(OH)_6_; Ghose, 1964 ▸), which is monoclinic. However, hydro­zincite does not have the same 1:1:1 ratio of metal:carbonate:hydroxide as the structure discussed in this article so an equivalent structure would not be expected.

Wei *et al.* (2021 ▸) report diffraction peak positions consistent with JCPDS card #41-0013. The defunct JCPDS database entries are now largely contained within the Inter­national Centre for Diffraction Data Powder Diffraction Files (ICDD-PDF) database. A search of ICDD-PDF for related structures revealed two entries #00-046-0369 and #00-043-0602 for Ce_2_(CO_3_)_2_(OH)_2_·H_2_O (D’Assuncao *et al.*, 1989 ▸) and Ce_2_O(CO_3_)_2_·H_2_O (Bentzen *et al.*, 1987 ▸), respectively. Both entries only include unit cell parameters from indexing, where the former phase was indexed based on similarity with Nd_2_(CO_3_)_2_(OH)_4_·xH_2_O. While the lattice parameters reported by Bentzen *et al.* (1987 ▸) are within 1% error of the values of the present study, the absence of a structure refinement and analogous lanthanoid oxy-carbonate hydrate structures leads us to believe that the structure they report is actually the hydroxide phase reported in the present work.

## 4. Experimental

Commercial ‘cerium(III) carbonate hydrate’ (REacton^TM^ grade 99.000%, lot: Y011E058) was purchased from Alfa Aesar. Synchrotron powder X-ray diffraction data were obtained at Stanford Synchrotron Radiation Lightsource (SSRL) beamline 2-1 (Stone *et al.*, 2023 ▸); wavelength was refined against a NIST 660C LaB_6_ standard. During the measurement, the sample was stored in a Nordson (141-0158) Kapton capillary with a 0.7112 mm outer diameter and 0.002 mm wall thickness.

## 5. Refinement

Structural data of the ortho­rhom­bic cerium(III) carbonate hydroxide title compound are given in Table 1 ▸. Diffraction data were fit by the Rietveld method (Rietveld, 1969 ▸, 2014 ▸) using *Topas (*Academic version 7; Coelho, 2018 ▸). Background was modelled using an order-13 Chebyshev polynomial; Bragg reflection peakshapes were modelled using the Thompson-Cox-Hastings pseudo-Voigt function (Thompson *et al.*, 1987 ▸); the approach of Stephens (1999 ▸) was used to model anisotropic peak broadening. As a result of their negligible X-ray scattering factor, H atoms were not located and thus are not included in any structural models in the Rietveld fit. For each site, isotropic atomic displacement parameters (*U*_iso_) were refined against the data, with *U*_iso_ for sites of the same element constrained to be equal.

## Supplementary Material

Crystal structure: contains datablock(s) I, CeriumIV_Oxide, CeriumIII_carbonate_hydroxide, Alfa_Aesar_CeIII_Carbonate. DOI: 10.1107/S2056989025003512/wm5755sup1.cif

CCDC references: 2445085, 2445084, 2445083

Additional supporting information:  crystallographic information; 3D view; checkCIF report

## Figures and Tables

**Table 1:** Experimental details

	Ce(CO_3_)(OH)
Crystal data	
*M*_r_	217.14
Crystal system, space group	Orthorhombic, *Pmcn*
Temperature (K)	300
*a*, *b*, *c* (Å)	5.01416 (15), 8.5569 (3), 7.3252 (2)
*V* (Å^3^)	314.29 (2)
*Z*	4
Radiation type	Synchrotron, λ = 0.729472 Å
μ (mm^−1^)	15.48
Specimen shape, size (mm)	Cylinder, 1 × 0.71

Data collection	
Diffractometer	SSRL beam line 2-1
Specimen mounting	Kapton Capillary
Data collection mode	Transmission
Scan method	Step
2θ values (°)	2θ_min_ = 0.01, 2θ_max_ = 116.605, 2θ_step_ = 0.005

Refinement	
*R* factors and goodness of fit	*R*_p_ = 2.574, *R*_wp_ = 3.583, *R*_exp_ = 15.749, χ^2^ = 0.052
No. of parameters	49

Computer programs: *spec* X-ray diffraction software (local program), *Topas 7* (Academic Version; Coelho, 2018 ▸), SSRL BL 2-1 Python integration script (Stone *et al.*, 2023 ▸), *VESTA-III* (Momma & Izumi, 2011 ▸) and *publCIF* (Westrip, 2010 ▸).

## supplementary crystallographic information

### Cerium(III) carbonate hydroxide (I) Crystal data

**Table:**

Ce(CO_3_)(OH)	*Z* = 4
*M**_r_* = 217.14	*D*_x_ = 4.568 Mg m^−^^3^
Orthorhombic, *P**m**c**n*	Synchrotron radiation, λ = 0.729472 Å
*a* = 5.01416 (15) Å	µ = 15.48 mm^−^^1^
*b* = 8.5569 (3) Å	*T* = 300 K
*c* = 7.3252 (2) Å	cylinder, 1 × 0.71 mm
*V* = 314.29 (2) Å^3^	

### Cerium(III) carbonate hydroxide (I) Data collection

**Table:**

SSRL beam line 2-1 diffractometer	Scan method: step
Specimen mounting: Kapton Capillary	2θ_min_ = 0.01°, 2θ_max_ = 116.605°, 2θ_step_ = 0.005°
Data collection mode: transmission	

### Cerium(III) carbonate hydroxide (I) Refinement

**Table:**

*R*_p_ = 2.574	49 parameters
*R*_wp_ = 3.583	0 restraints
*R*_exp_ = 15.749	

### Cerium(III) carbonate hydroxide (I) Fractional atomic coordinates and isotropic or equivalent isotropic displacement parameters (Å^2^)

**Table:**

	*x*	*y*	*z*	*U*_iso_*/*U*_eq_	
Ce1	0.25	0.33442 (4)	0.64574 (6)	0.00597 (8)	
C1	0.75	0.1810 (8)	0.8028 (8)	0.0182 (16)	
O1	0.75	0.3087 (4)	0.7166 (4)	0.0066 (5)	
O2	0.5236 (4)	0.1167 (2)	0.8416 (4)	0.0066 (5)	
O3	0.25	0.4027 (4)	0.9717 (5)	0.0066 (5)	

### Cerium(III) carbonate hydroxide (I) Geometric parameters (Å, º)

**Table:**

Ce1—O3^i^	2.396 (3)	O1—C1^xii^	3.033 (7)
Ce1—O3	2.458 (3)	O1—O2^xii^	3.040 (4)
Ce1—O1	2.5697 (8)	O1—O2^iii^	3.040 (4)
Ce1—O1^ii^	2.5697 (8)	O2—C1	1.293 (4)
Ce1—O2^iii^	2.650 (3)	O2—O1	2.197 (4)
Ce1—O2^i^	2.650 (3)	O2—O2^vii^	2.271 (4)
Ce1—O2^iv^	2.670 (2)	O2—Ce1^xiii^	2.650 (3)
Ce1—O2^v^	2.670 (2)	O2—Ce1^viii^	2.670 (2)
Ce1—O2	2.723 (2)	O2—Ce1	2.723 (2)
Ce1—O2^vi^	2.723 (2)	O2—O2^vi^	2.744 (4)
Ce1—C1^v^	2.989 (7)	O2—O3	2.963 (4)
Ce1—C1	3.055 (4)	O2—O1^viii^	3.001 (4)
Ce1—C1^ii^	3.055 (4)	O2—O1^ix^	3.040 (4)
C1—O1	1.262 (7)	O2—O3^i^	3.041 (4)
C1—O2^vii^	1.293 (4)	O2—O2^xiv^	3.071 (5)
C1—O2	1.293 (4)	O2—O3^viii^	3.148 (4)
C1—Ce1^viii^	2.989 (7)	O3—Ce1^xiii^	2.396 (3)
C1—O1^ix^	3.033 (7)	O3—Ce1	2.458 (3)
C1—Ce1^x^	3.055 (4)	O3—O2	2.963 (4)
C1—Ce1	3.055 (4)	O3—O2^vi^	2.963 (4)
C1—O3^viii^	3.117 (7)	O3—O3^xv^	3.038 (3)
O1—C1	1.262 (7)	O3—O3^xvi^	3.038 (3)
O1—O2^vii^	2.197 (4)	O3—O2^xiii^	3.041 (4)
O1—O2	2.197 (4)	O3—O2^xvii^	3.041 (4)
O1—Ce1^x^	2.5697 (8)	O3—C1^v^	3.117 (7)
O1—Ce1	2.5697 (8)	O3—O2^iv^	3.148 (4)
O1—O2^v^	3.001 (4)	O3—O2^v^	3.148 (4)
O1—O2^xi^	3.001 (4)		
			
O3—Ce1—O3^i^	135.89 (9)	O2^iii^—O1—O2^xii^	43.85 (10)
O1—Ce1—O3	79.87 (7)	O2^iii^—O1—C1^xii^	24.59 (8)
O1—Ce1—O3^i^	92.01 (8)	O2^iii^—O1—O2^xi^	81.83 (10)
O1^ii^—Ce1—O1	154.65 (14)	O2^iii^—O1—O2^v^	61.09 (10)
O1^ii^—Ce1—O3	79.87 (7)	O2^iii^—O1—Ce1	55.61 (6)
O1^ii^—Ce1—O3^i^	92.01 (8)	O2^iii^—O1—Ce1^x^	99.42 (10)
O2^iii^—Ce1—O1^ii^	133.51 (9)	O2^iii^—O1—O2	109.91 (12)
O2^iii^—Ce1—O1	71.24 (9)	O2^iii^—O1—O2^vii^	136.59 (17)
O2^iii^—Ce1—O3	141.19 (7)	O2^iii^—O1—C1	129.3 (3)
O2^iii^—Ce1—O3^i^	71.73 (8)	O1—O2—C1	30.3 (3)
O2^i^—Ce1—O2^iii^	62.36 (10)	O2^vii^—O2—O1	58.89 (7)
O2^i^—Ce1—O1^ii^	71.24 (9)	O2^vii^—O2—C1	28.6 (3)
O2^i^—Ce1—O1	133.51 (9)	Ce1^xiii^—O2—O2^vii^	121.18 (5)
O2^i^—Ce1—O3	141.19 (7)	Ce1^xiii^—O2—O1	119.97 (13)
O2^i^—Ce1—O3^i^	71.73 (8)	Ce1^xiii^—O2—C1	124.9 (3)
O2^iv^—Ce1—O2^i^	70.50 (9)	Ce1^viii^—O2—Ce1^xiii^	109.50 (9)
O2^iv^—Ce1—O2^iii^	96.11 (6)	Ce1^viii^—O2—O2^vii^	64.84 (5)
O2^iv^—Ce1—O1^ii^	69.85 (10)	Ce1^viii^—O2—O1	118.12 (11)
O2^iv^—Ce1—O1	119.02 (10)	Ce1^viii^—O2—C1	91.1 (3)
O2^iv^—Ce1—O3	75.60 (9)	Ce1—O2—Ce1^viii^	148.39 (11)
O2^iv^—Ce1—O3^i^	141.66 (8)	Ce1—O2—Ce1^xiii^	94.25 (7)
O2^v^—Ce1—O2^iv^	50.32 (9)	Ce1—O2—O2^vii^	120.26 (5)
O2^v^—Ce1—O2^i^	96.11 (6)	Ce1—O2—O1	61.89 (8)
O2^v^—Ce1—O2^iii^	70.50 (9)	Ce1—O2—C1	92.0 (3)
O2^v^—Ce1—O1^ii^	119.02 (10)	O2^vi^—O2—Ce1	59.74 (5)
O2^v^—Ce1—O1	69.85 (10)	O2^vi^—O2—Ce1^viii^	115.16 (5)
O2^v^—Ce1—O3	75.60 (9)	O2^vi^—O2—Ce1^xiii^	58.82 (5)
O2^v^—Ce1—O3^i^	141.66 (8)	O2^vi^—O2—O2^vii^	180.0000 (1)
O2—Ce1—O2^v^	112.74 (3)	O2^vi^—O2—O1	121.11 (7)
O2—Ce1—O2^iv^	144.58 (4)	O2^vi^—O2—C1	151.4 (3)
O2—Ce1—O2^i^	144.30 (6)	O3—O2—O2^vi^	62.42 (5)
O2—Ce1—O2^iii^	106.88 (7)	O3—O2—Ce1	51.02 (7)
O2—Ce1—O1^ii^	109.06 (9)	O3—O2—Ce1^viii^	158.85 (13)
O2—Ce1—O1	48.96 (9)	O3—O2—Ce1^xiii^	50.16 (7)
O2—Ce1—O3	69.56 (8)	O3—O2—O2^vii^	117.58 (5)
O2—Ce1—O3^i^	72.59 (9)	O3—O2—O1	75.84 (11)
O2^vi^—Ce1—O2	60.51 (9)	O3—O2—C1	97.2 (3)
O2^vi^—Ce1—O2^v^	144.58 (4)	O1^viii^—O2—O3	124.00 (8)
O2^vi^—Ce1—O2^iv^	112.74 (3)	O1^viii^—O2—O2^vi^	62.80 (5)
O2^vi^—Ce1—O2^i^	106.88 (7)	O1^viii^—O2—Ce1	107.20 (9)
O2^vi^—Ce1—O2^iii^	144.30 (6)	O1^viii^—O2—Ce1^viii^	53.50 (5)
O2^vi^—Ce1—O1^ii^	48.96 (9)	O1^viii^—O2—Ce1^xiii^	91.23 (8)
O2^vi^—Ce1—O1	109.06 (9)	O1^viii^—O2—O2^vii^	117.20 (5)
O2^vi^—Ce1—O3	69.56 (8)	O1^viii^—O2—O1	146.49 (13)
O2^vi^—Ce1—O3^i^	72.59 (9)	O1^viii^—O2—C1	138.1 (3)
C1^v^—Ce1—O2^vi^	127.77 (10)	O1^ix^—O2—O1^viii^	118.91 (10)
C1^v^—Ce1—O2	127.77 (10)	O1^ix^—O2—O3	73.06 (10)
C1^v^—Ce1—O2^v^	25.63 (5)	O1^ix^—O2—O2^vi^	111.93 (5)
C1^v^—Ce1—O2^iv^	25.63 (5)	O1^ix^—O2—Ce1	121.37 (10)
C1^v^—Ce1—O2^i^	87.09 (11)	O1^ix^—O2—Ce1^viii^	89.98 (10)
C1^v^—Ce1—O2^iii^	87.09 (11)	O1^ix^—O2—Ce1^xiii^	53.15 (6)
C1^v^—Ce1—O1^ii^	93.41 (9)	O1^ix^—O2—O2^vii^	68.07 (5)
C1^v^—Ce1—O1	93.41 (9)	O1^ix^—O2—O1	91.51 (10)
C1^v^—Ce1—O3	69.01 (14)	O1^ix^—O2—C1	77.4 (3)
C1^v^—Ce1—O3^i^	155.10 (14)	O3^i^—O2—O1^ix^	169.99 (11)
C1—Ce1—C1^v^	112.26 (11)	O3^i^—O2—O1^viii^	67.64 (10)
C1—Ce1—O2^vi^	85.47 (11)	O3^i^—O2—O3	97.05 (9)
C1—Ce1—O2	25.03 (11)	O3^i^—O2—O2^vi^	63.19 (5)
C1—Ce1—O2^v^	91.53 (12)	O3^i^—O2—Ce1	48.74 (7)
C1—Ce1—O2^iv^	136.43 (13)	O3^i^—O2—Ce1^viii^	100.00 (10)
C1—Ce1—O2^i^	144.04 (12)	O3^i^—O2—Ce1^xiii^	121.61 (8)
C1—Ce1—O2^iii^	87.69 (11)	O3^i^—O2—O2^vii^	116.81 (5)
C1—Ce1—O1^ii^	133.46 (12)	O3^i^—O2—O1	84.41 (12)
C1—Ce1—O1	24.00 (13)	O3^i^—O2—C1	102.9 (3)
C1—Ce1—O3	74.71 (12)	O2^xiv^—O2—O3^i^	127.11 (13)
C1—Ce1—O3^i^	80.59 (13)	O2^xiv^—O2—O1^ix^	58.82 (11)
C1^ii^—Ce1—C1	110.3 (2)	O2^xiv^—O2—O1^viii^	60.09 (9)
C1^ii^—Ce1—C1^v^	112.26 (11)	O2^xiv^—O2—O3	104.96 (14)
C1^ii^—Ce1—O2^vi^	25.03 (11)	O2^xiv^—O2—O2^vi^	85.58 (8)
C1^ii^—Ce1—O2	85.47 (11)	O2^xiv^—O2—Ce1	143.56 (12)
C1^ii^—Ce1—O2^v^	136.43 (13)	O2^xiv^—O2—Ce1^viii^	54.44 (6)
C1^ii^—Ce1—O2^iv^	91.53 (12)	O2^xiv^—O2—Ce1^xiii^	55.06 (9)
C1^ii^—Ce1—O2^i^	87.69 (11)	O2^xiv^—O2—O2^vii^	94.42 (8)
C1^ii^—Ce1—O2^iii^	144.04 (12)	O2^xiv^—O2—O1	147.24 (15)
C1^ii^—Ce1—O1^ii^	24.00 (13)	O2^xiv^—O2—C1	120.7 (3)
C1^ii^—Ce1—O1	133.46 (12)	O3^viii^—O2—O2^xiv^	101.58 (11)
C1^ii^—Ce1—O3	74.71 (12)	O3^viii^—O2—O3^i^	58.77 (8)
C1^ii^—Ce1—O3^i^	80.59 (13)	O3^viii^—O2—O1^ix^	130.26 (9)
O2^vii^—C1—O1	118.6 (3)	O3^viii^—O2—O1^viii^	63.29 (8)
O2—C1—O2^vii^	122.8 (5)	O3^viii^—O2—O3	151.88 (12)
O2—C1—O1	118.6 (3)	O3^viii^—O2—O2^vi^	111.14 (5)
Ce1^viii^—C1—O2	63.3 (3)	O3^viii^—O2—Ce1	101.29 (10)
Ce1^viii^—C1—O2^vii^	63.3 (3)	O3^viii^—O2—Ce1^viii^	49.15 (7)
Ce1^viii^—C1—O1	157.2 (4)	O3^viii^—O2—Ce1^xiii^	153.08 (10)
O1^ix^—C1—Ce1^viii^	84.42 (18)	O3^viii^—O2—O2^vii^	68.86 (5)
O1^ix^—C1—O2	78.0 (3)	O3^viii^—O2—O1	86.85 (13)
O1^ix^—C1—O2^vii^	78.0 (3)	O3^viii^—O2—C1	76.8 (3)
O1^ix^—C1—O1	118.3 (4)	Ce1—O3—Ce1^xiii^	108.40 (12)
Ce1^x^—C1—O1^ix^	111.34 (14)	O2—O3—Ce1	59.42 (9)
Ce1^x^—C1—Ce1^viii^	118.28 (13)	O2—O3—Ce1^xiii^	58.12 (8)
Ce1^x^—C1—O2	170.4 (4)	O2^vi^—O3—O2	55.15 (10)
Ce1^x^—C1—O2^vii^	62.95 (18)	O2^vi^—O3—Ce1	59.42 (9)
Ce1^x^—C1—O1	55.91 (11)	O2^vi^—O3—Ce1^xiii^	58.12 (8)
Ce1—C1—Ce1^x^	110.3 (2)	O3^xv^—O3—O2^vi^	96.59 (9)
Ce1—C1—O1^ix^	111.34 (14)	O3^xv^—O3—O2	151.48 (15)
Ce1—C1—Ce1^viii^	118.28 (13)	O3^xv^—O3—Ce1	105.23 (13)
Ce1—C1—O2	62.95 (18)	O3^xv^—O3—Ce1^xiii^	113.06 (13)
Ce1—C1—O2^vii^	170.4 (4)	O3^xvi^—O3—O3^xv^	111.2 (2)
Ce1—C1—O1	55.91 (11)	O3^xvi^—O3—O2^vi^	151.48 (15)
O3^viii^—C1—Ce1	94.90 (14)	O3^xvi^—O3—O2	96.59 (9)
O3^viii^—C1—Ce1^x^	94.90 (14)	O3^xvi^—O3—Ce1	105.23 (13)
O3^viii^—C1—O1^ix^	131.8 (2)	O3^xvi^—O3—Ce1^xiii^	113.06 (13)
O3^viii^—C1—Ce1^viii^	47.42 (12)	O2^xiii^—O3—O3^xvi^	106.29 (16)
O3^viii^—C1—O2	79.4 (3)	O2^xiii^—O3—O3^xv^	62.36 (11)
O3^viii^—C1—O2^vii^	79.4 (3)	O2^xiii^—O3—O2^vi^	91.88 (9)
O3^viii^—C1—O1	109.8 (4)	O2^xiii^—O3—O2	116.78 (12)
O2^vii^—O1—C1	31.12 (8)	O2^xiii^—O3—Ce1	148.48 (9)
O2—O1—O2^vii^	62.22 (15)	O2^xiii^—O3—Ce1^xiii^	58.67 (8)
O2—O1—C1	31.12 (8)	O2^xvii^—O3—O2^xiii^	53.62 (10)
Ce1^x^—O1—O2	130.71 (14)	O2^xvii^—O3—O3^xvi^	62.36 (11)
Ce1^x^—O1—O2^vii^	69.15 (7)	O2^xvii^—O3—O3^xv^	106.29 (16)
Ce1^x^—O1—C1	100.09 (9)	O2^xvii^—O3—O2^vi^	116.78 (12)
Ce1—O1—Ce1^x^	154.65 (14)	O2^xvii^—O3—O2	91.88 (9)
Ce1—O1—O2	69.15 (7)	O2^xvii^—O3—Ce1	148.48 (9)
Ce1—O1—O2^vii^	130.71 (14)	O2^xvii^—O3—Ce1^xiii^	58.67 (8)
Ce1—O1—C1	100.09 (9)	C1^v^—O3—O2^xvii^	128.04 (14)
O2^v^—O1—Ce1	56.65 (6)	C1^v^—O3—O2^xiii^	128.04 (14)
O2^v^—O1—Ce1^x^	110.01 (12)	C1^v^—O3—O3^xvi^	70.68 (13)
O2^v^—O1—O2	118.67 (5)	C1^v^—O3—O3^xv^	70.68 (13)
O2^v^—O1—O2^vii^	162.19 (15)	C1^v^—O3—O2^vi^	115.06 (15)
O2^v^—O1—C1	146.2 (2)	C1^v^—O3—O2	115.06 (15)
O2^xi^—O1—O2^v^	54.40 (10)	C1^v^—O3—Ce1	63.57 (14)
O2^xi^—O1—Ce1	110.01 (12)	C1^v^—O3—Ce1^xiii^	171.97 (18)
O2^xi^—O1—Ce1^x^	56.65 (6)	O2^iv^—O3—C1^v^	23.82 (7)
O2^xi^—O1—O2	162.19 (15)	O2^iv^—O3—O2^xvii^	147.54 (12)
O2^xi^—O1—O2^vii^	118.67 (5)	O2^iv^—O3—O2^xiii^	121.23 (8)
O2^xi^—O1—C1	146.2 (2)	O2^iv^—O3—O3^xvi^	94.48 (15)
C1^xii^—O1—O2^xi^	80.36 (15)	O2^iv^—O3—O3^xv^	58.86 (11)
C1^xii^—O1—O2^v^	80.36 (15)	O2^iv^—O3—O2^vi^	94.53 (8)
C1^xii^—O1—Ce1	78.20 (7)	O2^iv^—O3—O2	114.38 (11)
C1^xii^—O1—Ce1^x^	78.20 (7)	O2^iv^—O3—Ce1	55.25 (8)
C1^xii^—O1—O2	115.98 (17)	O2^iv^—O3—Ce1^xiii^	151.69 (10)
C1^xii^—O1—O2^vii^	115.98 (17)	O2^v^—O3—O2^iv^	42.28 (9)
C1^xii^—O1—C1	121.7 (3)	O2^v^—O3—C1^v^	23.82 (7)
O2^xii^—O1—C1^xii^	24.59 (8)	O2^v^—O3—O2^xvii^	121.23 (8)
O2^xii^—O1—O2^xi^	61.09 (10)	O2^v^—O3—O2^xiii^	147.54 (12)
O2^xii^—O1—O2^v^	81.83 (10)	O2^v^—O3—O3^xvi^	58.86 (11)
O2^xii^—O1—Ce1	99.42 (10)	O2^v^—O3—O3^xv^	94.48 (15)
O2^xii^—O1—Ce1^x^	55.61 (6)	O2^v^—O3—O2^vi^	114.38 (11)
O2^xii^—O1—O2	136.59 (17)	O2^v^—O3—O2	94.53 (8)
O2^xii^—O1—O2^vii^	109.91 (12)	O2^v^—O3—Ce1	55.25 (8)
O2^xii^—O1—C1	129.3 (3)	O2^v^—O3—Ce1^xiii^	151.69 (10)

Symmetry codes: (i) −*x*+1/2, −*y*+1/2, *z*−1/2; (ii) *x*−1, *y*, *z*; (iii) *x*, −*y*+1/2, *z*−1/2; (iv) *x*−1/2, *y*+1/2, −*z*+3/2; (v) −*x*+1, *y*+1/2, −*z*+3/2; (vi) −*x*+1/2, *y*, *z*; (vii) −*x*+3/2, *y*, *z*; (viii) −*x*+1, *y*−1/2, −*z*+3/2; (ix) −*x*+3/2, −*y*+1/2, *z*+1/2; (x) *x*+1, *y*, *z*; (xi) *x*+1/2, *y*+1/2, −*z*+3/2; (xii) −*x*+3/2, −*y*+1/2, *z*−1/2; (xiii) −*x*+1/2, −*y*+1/2, *z*+1/2; (xiv) −*x*+1, −*y*, −*z*+2; (xv) −*x*, −*y*+1, −*z*+2; (xvi) −*x*+1, −*y*+1, −*z*+2; (xvii) *x*, −*y*+1/2, *z*+1/2.

### (CeriumIV_Oxide) Crystal data

**Table:**

CeO_2_	*D*_x_ = 7.151 Mg m^−^^3^
*M**_r_* = 172.11	Synchrotron radiation, λ = 0.729472 Å
Cubic, *F**m*3*m*	µ = 30.22 mm^−^^1^
*a* = 5.42732 (16) Å	*T* = 300 K
*V* = 159.87 (1) Å^3^	cylinder, 1 × 0.71 mm
*Z* = 4	

### (CeriumIV_Oxide) Data collection

**Table:**

SSRL beam line 2-1 diffractometer	Scan method: step
Specimen mounting: Kapton Capillary	2θ_min_ = 0.01°, 2θ_max_ = 116.61°, 2θ_step_ = 0.005°
Data collection mode: transmission	

### (CeriumIV_Oxide) Refinement

**Table:**

*R*_p_ = 2.574	49 parameters
*R*_wp_ = 3.583	0 restraints
*R*_exp_ = 15.749	

### (CeriumIV_Oxide) Fractional atomic coordinates and isotropic or equivalent isotropic displacement parameters (Å^2^)

**Table:**

	*x*	*y*	*z*	*U*_iso_*/*U*_eq_	
Ce1	0	0	0	0.00595 (6)	
O1	0.25	0.25	0.25	0.0115 (4)	

### (CeriumIV_Oxide) Geometric parameters (Å, º)

**Table:**

Ce1—O1^i^	2.3501 (1)	O1—Ce1^ix^	2.3501 (1)
Ce1—O1^ii^	2.3501 (1)	O1—Ce1^x^	2.3501 (1)
Ce1—O1^iii^	2.3501 (1)	O1—Ce1	2.3501 (1)
Ce1—O1^iv^	2.3501 (1)	O1—O1^xi^	2.7137 (1)
Ce1—O1^v^	2.3501 (1)	O1—O1^xii^	2.7137 (1)
Ce1—O1^vi^	2.3501 (1)	O1—O1^xiii^	2.7137 (1)
Ce1—O1	2.3501 (1)	O1—O1^i^	2.7137 (1)
Ce1—O1^vii^	2.3501 (1)	O1—O1^vi^	2.7137 (1)
O1—Ce1^viii^	2.3501 (1)	O1—O1^iii^	2.7137 (1)
			
O1^ii^—Ce1—O1^i^	70.529	O1^xi^—O1—Ce1^viii^	54.736
O1^iii^—Ce1—O1^ii^	180.000	O1^xii^—O1—O1^xi^	90.000
O1^iii^—Ce1—O1^i^	109.471	O1^xii^—O1—Ce1	125.264
O1^iv^—Ce1—O1^iii^	70.529	O1^xii^—O1—Ce1^x^	125.264
O1^iv^—Ce1—O1^ii^	109.471	O1^xii^—O1—Ce1^ix^	54.736
O1^iv^—Ce1—O1^i^	70.529	O1^xii^—O1—Ce1^viii^	54.736
O1^v^—Ce1—O1^iv^	109.471	O1^xiii^—O1—O1^xii^	90.000
O1^v^—Ce1—O1^iii^	70.529	O1^xiii^—O1—O1^xi^	90.000
O1^v^—Ce1—O1^ii^	109.471	O1^xiii^—O1—Ce1	125.264
O1^v^—Ce1—O1^i^	180.000	O1^xiii^—O1—Ce1^x^	54.736
O1^vi^—Ce1—O1^v^	70.529	O1^xiii^—O1—Ce1^ix^	54.736
O1^vi^—Ce1—O1^iv^	180.000	O1^xiii^—O1—Ce1^viii^	125.264
O1^vi^—Ce1—O1^iii^	109.471	O1^i^—O1—O1^xiii^	180.000
O1^vi^—Ce1—O1^ii^	70.529	O1^i^—O1—O1^xii^	90.000
O1^vi^—Ce1—O1^i^	109.471	O1^i^—O1—O1^xi^	90.000
O1—Ce1—O1^vi^	70.529	O1^i^—O1—Ce1	54.736
O1—Ce1—O1^v^	109.471	O1^i^—O1—Ce1^x^	125.264
O1—Ce1—O1^iv^	109.471	O1^i^—O1—Ce1^ix^	125.264
O1—Ce1—O1^iii^	70.529	O1^i^—O1—Ce1^viii^	54.736
O1—Ce1—O1^ii^	109.471	O1^vi^—O1—O1^i^	90.000
O1—Ce1—O1^i^	70.529	O1^vi^—O1—O1^xiii^	90.000
O1^vii^—Ce1—O1	180.000	O1^vi^—O1—O1^xii^	180.000
O1^vii^—Ce1—O1^vi^	109.471	O1^vi^—O1—O1^xi^	90.000
O1^vii^—Ce1—O1^v^	70.529	O1^vi^—O1—Ce1	54.736
O1^vii^—Ce1—O1^iv^	70.529	O1^vi^—O1—Ce1^x^	54.736
O1^vii^—Ce1—O1^iii^	109.471	O1^vi^—O1—Ce1^ix^	125.264
O1^vii^—Ce1—O1^ii^	70.529	O1^vi^—O1—Ce1^viii^	125.264
O1^vii^—Ce1—O1^i^	109.471	O1^iii^—O1—O1^vi^	90.000
Ce1^ix^—O1—Ce1^viii^	109.471	O1^iii^—O1—O1^i^	90.000
Ce1^x^—O1—Ce1^ix^	109.471	O1^iii^—O1—O1^xiii^	90.000
Ce1^x^—O1—Ce1^viii^	109.471	O1^iii^—O1—O1^xii^	90.000
Ce1—O1—Ce1^x^	109.471	O1^iii^—O1—O1^xi^	180.000
Ce1—O1—Ce1^ix^	109.471	O1^iii^—O1—Ce1	54.736
Ce1—O1—Ce1^viii^	109.471	O1^iii^—O1—Ce1^x^	125.264
O1^xi^—O1—Ce1	125.264	O1^iii^—O1—Ce1^ix^	54.736
O1^xi^—O1—Ce1^x^	54.736	O1^iii^—O1—Ce1^viii^	125.264
O1^xi^—O1—Ce1^ix^	125.264		

Symmetry codes: (i) −*x*, *z*, *y*; (ii) −*x*, *z*, −*y*; (iii) *z*, −*x*, *y*; (iv) −*x*, −*y*, *z*; (v) *z*, −*x*, −*y*; (vi) *z*, *y*, −*x*; (vii) −*x*, −*z*, −*y*; (viii) *x*, *y*+1/2, *z*+1/2; (ix) *x*+1/2, *y*, *z*+1/2; (x) *x*+1/2, *y*+1/2, *z*; (xi) *z*, −*x*+1, *y*; (xii) *z*, *y*, −*x*+1; (xiii) −*x*+1, *z*, *y*.

### (CeriumIII_carbonate_hydroxide) Crystal data

**Table:**

CeCHO_4_	*Z* = 18
*M**_r_* = 217.13	*D*_x_ = 4.925 Mg m^−^^3^
Hexagonal, *P*6	Synchrotron radiation, λ = 0.729472 Å
*a* = 12.5550 (12) Å	µ = 16.18 mm^−^^1^
*c* = 9.9257 (15) Å	*T* = 300 K
*V* = 1355.0 (3) Å^3^	cylinder, 1 × 0.71 mm

### (CeriumIII_carbonate_hydroxide) Data collection

**Table:**

SSRL beam line 2-1 diffractometer	Scan method: step
Specimen mounting: Kapton Capillary	2θ_min_ = 0.01°, 2θ_max_ = 116.605°, 2θ_step_ = 0.005°
Data collection mode: transmission	

### (CeriumIII_carbonate_hydroxide) Refinement

**Table:**

*R*_p_ = 2.574	49 parameters
*R*_wp_ = 3.583	0 restraints
*R*_exp_ = 15.749	

### (CeriumIII_carbonate_hydroxide) Fractional atomic coordinates and isotropic or equivalent isotropic displacement parameters (Å^2^)

**Table:**

	*x*	*y*	*z*	*U*_iso_*/*U*_eq_	
Ce1	0.10783	0.22796	0.2353	0.009010	
Ce2	0.77882	0.56268	0.2542	0.013740	
Ce3	0.43856	0.54375	0.25769	0.010700	
C1	0.8655	0.5103	0	0.030000	
C2	−0.0902	0.1104	0	0.007000	
C3	0.3037	0.4637	0	0.025000	
C4	0.5531	0.4206	0.5	0.026000	
C5	0.5356	0.7746	0.5	0.017000	
C6	−0.0247	0.1777	0.5	0.016000	
O1	0.7601	0.5088	0	0.034000	
O2	0.9117	0.5098	0.1118	0.017800	
O3	0.0286	0.1691	0	0.016800	
O4	0.2316	0.1469	0.1119	0.013900	
O5	0.4114	0.5726	0	0.040000	
O6	0.2516	0.4173	0.1117	0.025600	
O7	0.8033	0.5032	0.5	0.030000	
O8	0.5796	0.4838	0.3884	0.018600	
O9	0.3638	0.5249	0.5	0.033000	
O10	0.5671	0.7415	0.3879	0.021800	
O11	0.0822	0.1794	0.5	0.004300	
O12	−0.0721	0.1799	0.3875	0.014200	
O13	0	0	0.2723	0.009100	
O14	0.6667	0.3333	0.2377	0.027000	
O15	0.3333	0.6667	0.2356	0.023000	
O16	0.9998	0.6804	0.3218	0.016800	
O17	0.6532	0.6543	0.1778	0.020400	

### (CeriumIII_carbonate_hydroxide) Geometric parameters (Å, º)

**Table:**

Ce1—O16^i^	2.4641 (2)	O5—O6^iv^	3.0958 (3)
Ce1—O6	2.4745 (2)	O6—C3	1.2710 (1)
Ce1—O3	2.5010 (3)	O6—O6^vi^	2.2174 (3)
Ce1—O13	2.5068 (2)	O6—O5	2.2681 (2)
Ce1—O17^ii^	2.5193 (2)	O6—Ce1	2.4745 (2)
Ce1—O12	2.5271 (2)	O6—Ce3	2.5305 (2)
Ce1—O4	2.5558 (2)	O6—O16^i^	2.7637 (3)
Ce1—O11	2.6799 (4)	O6—O17^ii^	2.8847 (3)
Ce1—O10^ii^	2.7318 (2)	O6—O10^ii^	2.9468 (4)
Ce1—C6	3.0033 (4)	O6—O15^iv^	3.0250 (2)
Ce1—O4^iii^	3.0350 (2)	O6—O15^ii^	3.0259 (2)
Ce1—C2	3.1851 (3)	O6—O15	3.0261 (2)
Ce2—O17	2.4899 (2)	O6—O5^ii^	3.0958 (3)
Ce2—O12^iv^	2.4954 (2)	O6—O2^i^	3.0968 (3)
Ce2—O16	2.4963 (2)	O6—O3	3.1708 (3)
Ce2—O14^i^	2.4985 (2)	O7—C4^v^	1.3111 (1)
Ce2—O14^v^	2.4996 (2)	O7—O8^v^	2.2868 (2)
Ce2—O14	2.4996 (2)	O7—O8^xx^	2.2868 (2)
Ce2—O2	2.5158 (2)	O7—Ce2	2.6142 (3)
Ce2—O8	2.5562 (2)	O7—Ce2^xi^	2.6142 (3)
Ce2—O1	2.5923 (4)	O7—C4	2.7722 (3)
Ce2—O7	2.6142 (3)	O7—O8^xi^	2.9138 (2)
Ce2—O4^v^	2.6789 (2)	O7—O8	2.9138 (2)
Ce2—C1	2.9493 (3)	O7—O16	2.9455 (2)
Ce3—O17	2.4651 (2)	O7—O16^xi^	2.9455 (2)
Ce3—O15^iv^	2.4923 (2)	O7—O12^iv^	3.1764 (3)
Ce3—O15^ii^	2.4924 (2)	O7—O12^xiii^	3.1764 (3)
Ce3—O15	2.4935 (2)	O8—C4	1.3051 (1)
Ce3—O16^i^	2.5224 (2)	O8—O8^xi^	2.2154 (3)
Ce3—O6	2.5305 (2)	O8—O7^xii^	2.2868 (2)
Ce3—O10	2.5362 (2)	O8—Ce2	2.5562 (2)
Ce3—O9	2.5494 (3)	O8—Ce3	2.5919 (2)
Ce3—O8	2.5919 (2)	O8—O17	2.7978 (3)
Ce3—O2^i^	2.6160 (2)	O8—O7	2.9138 (2)
Ce3—O5	2.6293 (4)	O8—O16^i^	2.9352 (3)
Ce3—C3	2.9525 (3)	O8—O2^i^	2.9497 (4)
Ce3—O10^ii^	3.1608 (3)	O8—O14^i^	3.0106 (2)
C1—O2^vi^	1.2536 (1)	O8—O14^v^	3.0108 (2)
C1—O2	1.2536 (1)	O8—O14	3.0116 (2)
C1—O1	1.3140 (1)	O8—O12^iv^	3.1787 (3)
C1—O1^v^	2.9491 (3)	O8—O9	3.1988 (3)
C1—Ce2	2.9493 (3)	O9—C5^ii^	1.3572 (1)
C1—Ce2^vi^	2.9493 (3)	O9—O10^xvii^	2.3410 (2)
C1—O4^vii^	3.1779 (3)	O9—O10^ii^	2.3410 (2)
C1—O4^v^	3.1779 (3)	O9—Ce3	2.5494 (3)
C2—O4^iii^	1.2708 (1)	O9—Ce3^xi^	2.5494 (3)
C2—O4^viii^	1.2708 (1)	O9—C5	2.7782 (3)
C2—O3	1.2917 (1)	O9—O10	2.8648 (2)
C2—O3^viii^	2.7899 (3)	O9—O10^xi^	2.8648 (2)
C2—O17^ix^	3.1361 (2)	O9—O16^xii^	2.9430 (2)
C2—O17^ii^	3.1361 (2)	O9—O16^i^	2.9430 (2)
C2—O1^ii^	3.1659 (3)	O9—O8	3.1988 (3)
C2—Ce1^vi^	3.1851 (3)	O9—O8^xi^	3.1988 (3)
C2—Ce1	3.1851 (3)	O10—C5	1.3159 (1)
C3—O6	1.2710 (1)	O10—O10^xi^	2.2253 (3)
C3—O6^vi^	1.2710 (1)	O10—O9^xiii^	2.3410 (2)
C3—O5	1.3598 (1)	O10—Ce3	2.5362 (2)
C3—O5^ii^	2.9095 (3)	O10—Ce1^iv^	2.7318 (2)
C3—Ce3^vi^	2.9525 (3)	O10—O17	2.8106 (3)
C3—Ce3	2.9525 (3)	O10—O9	2.8648 (2)
C3—O2^x^	3.0221 (2)	O10—O16^xiv^	2.8952 (3)
C3—O2^i^	3.0221 (2)	O10—O6^iv^	2.9468 (4)
C4—O8	1.3051 (1)	O10—O15^ii^	3.0034 (2)
C4—O8^xi^	1.3051 (1)	O10—O11^xiii^	3.0039 (2)
C4—O7^xii^	1.3111 (1)	O10—O15^iv^	3.0042 (2)
C4—O7	2.7722 (3)	O10—O15	3.0045 (2)
C4—O16^xii^	3.1004 (2)	O10—C6^xiii^	3.1214 (3)
C4—O16^i^	3.1004 (2)	O10—O12^iv^	3.1364 (3)
C5—O10^xi^	1.3159 (1)	O10—Ce3^iv^	3.1608 (3)
C5—O10	1.3159 (1)	O11—C6	1.3316 (1)
C5—O9^xiii^	1.3572 (1)	O11—O12^xi^	2.2387 (2)
C5—O9	2.7782 (3)	O11—O12	2.2387 (2)
C5—O16^xiv^	3.0510 (2)	O11—Ce1	2.6799 (4)
C5—O16^xv^	3.0510 (2)	O11—Ce1^xi^	2.6799 (4)
C5—O11^xiii^	3.1040 (3)	O11—O13^xvi^	2.9869 (3)
C6—O12^xi^	1.2721 (1)	O11—O13	2.9869 (3)
C6—O12	1.2721 (1)	O11—C6^xix^	2.9968 (3)
C6—O11	1.3316 (1)	O11—O10^ii^	3.0039 (2)
C6—O11^xvi^	2.9968 (3)	O11—O10^xvii^	3.0039 (2)
C6—Ce1	3.0033 (4)	O11—C5^ii^	3.1040 (3)
C6—Ce1^xi^	3.0033 (4)	O11—O16^i^	3.1405 (2)
C6—O10^ii^	3.1214 (3)	O11—O16^xii^	3.1405 (2)
C6—O10^xvii^	3.1214 (3)	O12—C6	1.2721 (1)
O1—C1	1.3140 (1)	O12—O12^xi^	2.2333 (3)
O1—O2^vi^	2.1978 (2)	O12—O11	2.2387 (2)
O1—O2	2.1978 (2)	O12—Ce2^ii^	2.4954 (2)
O1—Ce2^vi^	2.5923 (4)	O12—Ce1	2.5271 (2)
O1—Ce2	2.5923 (4)	O12—O17^ii^	2.7644 (3)
O1—C1^x^	2.9491 (3)	O12—O16^ii^	2.8549 (3)
O1—O14^i^	3.0346 (3)	O12—O4^iii^	2.9447 (4)
O1—O14^x^	3.0346 (3)	O12—O13	3.0451 (2)
O1—O14	3.0353 (3)	O12—O10^ii^	3.1364 (3)
O1—O14^vi^	3.0353 (3)	O12—O7^ii^	3.1764 (3)
O1—O14^v^	3.0353 (3)	O12—O8^ii^	3.1787 (3)
O1—O14^vii^	3.0353 (3)	O13—Ce1^xix^	2.5068 (2)
O1—O4^vii^	3.0500 (3)	O13—Ce1	2.5068 (2)
O1—O4^v^	3.0500 (3)	O13—Ce1^iii^	2.5068 (2)
O1—O2^i^	3.1571 (3)	O13—O11^xvi^	2.9869 (3)
O1—O2^x^	3.1571 (3)	O13—O11^xix^	2.9869 (3)
O1—C2^xviii^	3.1659 (3)	O13—O11	2.9869 (3)
O2—C1	1.2536 (1)	O13—O4	3.0047 (2)
O2—O1	2.1978 (2)	O13—O4^iii^	3.0047 (2)
O2—O2^vi^	2.2194 (3)	O13—O4^xix^	3.0047 (2)
O2—Ce2	2.5158 (2)	O13—O12^iii^	3.0451 (2)
O2—Ce3^v^	2.6160 (2)	O13—O12	3.0451 (2)
O2—O16	2.7905 (3)	O13—O12^xix^	3.0451 (2)
O2—O17^v^	2.8590 (3)	O14—Ce2^v^	2.4985 (2)
O2—O8^v^	2.9497 (4)	O14—Ce2^i^	2.4996 (2)
O2—O5^v^	2.9872 (2)	O14—Ce2	2.4996 (2)
O2—O14^i^	3.0192 (2)	O14—O8^v^	3.0106 (2)
O2—O14	3.0195 (2)	O14—O8^i^	3.0108 (2)
O2—O14^v^	3.0203 (2)	O14—O8	3.0116 (2)
O2—C3^v^	3.0221 (2)	O14—O2^v^	3.0192 (2)
O2—O6^v^	3.0968 (3)	O14—O2	3.0195 (2)
O2—O1^v^	3.1571 (3)	O14—O2^i^	3.0203 (2)
O3—C2	1.2917 (1)	O14—O1^v^	3.0346 (3)
O3—O4^iii^	2.2083 (2)	O14—O1	3.0353 (3)
O3—O4^viii^	2.2083 (2)	O14—O1^x^	3.0353 (3)
O3—Ce1^vi^	2.5010 (3)	O15—Ce3^ii^	2.4923 (2)
O3—Ce1	2.5010 (3)	O15—Ce3^iv^	2.4924 (2)
O3—C2^xix^	2.7899 (3)	O15—Ce3	2.4935 (2)
O3—O4^vi^	2.9185 (2)	O15—O5^ii^	2.9967 (3)
O3—O4	2.9185 (2)	O15—O5^xviii^	2.9967 (3)
O3—O17^ix^	2.9969 (2)	O15—O5	2.9974 (3)
O3—O17^ii^	2.9969 (2)	O15—O10^iv^	3.0034 (2)
O3—O6^vi^	3.1708 (3)	O15—O10^ii^	3.0042 (2)
O3—O6	3.1708 (3)	O15—O10	3.0045 (2)
O4—C2^xix^	1.2708 (1)	O15—O6^ii^	3.0250 (2)
O4—O3^xix^	2.2083 (2)	O15—O6^iv^	3.0259 (2)
O4—O4^vi^	2.2214 (3)	O15—O6	3.0261 (2)
O4—Ce1	2.5558 (2)	O16—Ce1^v^	2.4641 (2)
O4—Ce2^i^	2.6789 (2)	O16—Ce2	2.4963 (2)
O4—O16^i^	2.8034 (3)	O16—Ce3^v^	2.5224 (2)
O4—O3	2.9185 (2)	O16—O6^v^	2.7637 (3)
O4—O17^i^	2.9316 (3)	O16—O2	2.7905 (3)
O4—O12^xix^	2.9447 (4)	O16—O4^v^	2.8034 (3)
O4—O13	3.0047 (2)	O16—O12^iv^	2.8549 (3)
O4—Ce1^xix^	3.0350 (2)	O16—O10^xxi^	2.8952 (3)
O4—O1^x^	3.0500 (3)	O16—O8^v^	2.9352 (3)
O4—C1^x^	3.1779 (3)	O16—O9^v^	2.9430 (2)
O5—C3	1.3598 (1)	O16—O7	2.9455 (2)
O5—O6	2.2681 (2)	O16—C5^xxii^	3.0510 (2)
O5—O6^vi^	2.2681 (2)	O16—C4^v^	3.1004 (2)
O5—Ce3^vi^	2.6293 (4)	O16—O11^v^	3.1405 (2)
O5—Ce3	2.6293 (4)	O17—Ce3	2.4651 (2)
O5—C3^xviii^	2.9095 (3)	O17—Ce2	2.4899 (2)
O5—O2^i^	2.9872 (2)	O17—Ce1^iv^	2.5193 (2)
O5—O2^x^	2.9872 (2)	O17—O12^iv^	2.7644 (3)
O5—O15^xviii^	2.9967 (3)	O17—O8	2.7978 (3)
O5—O15^iv^	2.9967 (3)	O17—O10	2.8106 (3)
O5—O15^ix^	2.9967 (3)	O17—O2^i^	2.8590 (3)
O5—O15^ii^	2.9967 (3)	O17—O6^iv^	2.8847 (3)
O5—O15^vi^	2.9974 (3)	O17—O4^v^	2.9316 (3)
O5—O15	2.9974 (3)	O17—O3^xviii^	2.9969 (2)
O5—O6^xviii^	3.0958 (3)	O17—C2^xviii^	3.1361 (2)
			
O6—Ce1—O16^i^	68.056 (5)	O15^iv^—O6—Ce1	119.969 (7)
O3—Ce1—O6	79.175 (7)	O15^iv^—O6—O5	67.212 (6)
O3—Ce1—O16^i^	129.8011 (2)	O15^iv^—O6—O6^vi^	113.988 (4)
O13—Ce1—O3	83.771 (4)	O15^iv^—O6—C3	92.857 (7)
O13—Ce1—O6	152.565 (2)	O15^ii^—O6—O15^iv^	0.0173 (1)
O13—Ce1—O16^i^	108.7737 (16)	O15^ii^—O6—O10^ii^	60.385 (3)
O17^ii^—Ce1—O13	124.4587 (3)	O15^ii^—O6—O17^ii^	88.7935 (18)
O17^ii^—Ce1—O3	73.3033 (17)	O15^ii^—O6—O16^i^	97.647 (7)
O17^ii^—Ce1—O6	70.5643 (14)	O15^ii^—O6—Ce3	52.383 (2)
O17^ii^—Ce1—O16^i^	124.9270 (7)	O15^ii^—O6—Ce1	119.982 (7)
O12—Ce1—O17^ii^	66.432 (5)	O15^ii^—O6—O5	67.196 (6)
O12—Ce1—O13	74.4448 (8)	O15^ii^—O6—O6^vi^	113.981 (4)
O12—Ce1—O3	108.161 (8)	O15^ii^—O6—C3	92.844 (7)
O12—Ce1—O6	131.403 (3)	O15—O6—O15^ii^	0.0234 (1)
O12—Ce1—O16^i^	122.034 (8)	O15—O6—O15^iv^	0.0119 (1)
O4—Ce1—O12	147.1714 (15)	O15—O6—O10^ii^	60.378 (3)
O4—Ce1—O17^ii^	137.442 (6)	O15—O6—O17^ii^	88.7713 (18)
O4—Ce1—O13	72.806 (2)	O15—O6—O16^i^	97.659 (7)
O4—Ce1—O3	70.489 (6)	O15—O6—Ce3	52.405 (2)
O4—Ce1—O6	81.271 (4)	O15—O6—Ce1	119.973 (7)
O4—Ce1—O16^i^	67.869 (5)	O15—O6—O5	67.210 (6)
O11—Ce1—O4	114.016 (5)	O15—O6—O6^vi^	113.979 (4)
O11—Ce1—O12	50.824 (4)	O15—O6—C3	92.851 (7)
O11—Ce1—O17^ii^	108.5367 (13)	O5^ii^—O6—O15	58.603 (5)
O11—Ce1—O13	70.2311 (5)	O5^ii^—O6—O15^ii^	58.620 (5)
O11—Ce1—O3	149.713 (5)	O5^ii^—O6—O15^iv^	58.615 (5)
O11—Ce1—O6	130.658 (3)	O5^ii^—O6—O10^ii^	92.292 (6)
O11—Ce1—O16^i^	75.123 (4)	O5^ii^—O6—O17^ii^	64.688 (3)
O10^ii^—Ce1—O11	67.423 (7)	O5^ii^—O6—O16^i^	151.7995 (16)
O10^ii^—Ce1—O4	132.857 (2)	O5^ii^—O6—Ce3	108.452 (3)
O10^ii^—Ce1—O12	73.108 (5)	O5^ii^—O6—Ce1	120.015 (2)
O10^ii^—Ce1—O17^ii^	64.572 (5)	O5^ii^—O6—O5	72.644 (3)
O10^ii^—Ce1—O13	136.916 (6)	O5^ii^—O6—O6^vi^	69.014 (3)
O10^ii^—Ce1—O3	133.255 (3)	O5^ii^—O6—C3	69.672 (3)
O10^ii^—Ce1—O6	68.738 (8)	O2^i^—O6—O5^ii^	138.2089 (15)
O10^ii^—Ce1—O16^i^	67.498 (2)	O2^i^—O6—O15	103.0890 (4)
C6—Ce1—O10^ii^	65.751 (6)	O2^i^—O6—O15^ii^	103.0656 (4)
C6—Ce1—O11	26.318 (4)	O2^i^—O6—O15^iv^	103.0804 (4)
C6—Ce1—O4	135.9611 (18)	O2^i^—O6—O10^ii^	111.373 (3)
C6—Ce1—O12	24.7246 (6)	O2^i^—O6—O17^ii^	157.0762 (13)
C6—Ce1—O17^ii^	85.397 (5)	O2^i^—O6—O16^i^	56.525 (4)
C6—Ce1—O13	72.8880 (3)	O2^i^—O6—Ce3	54.281 (2)
C6—Ce1—O3	131.157 (8)	O2^i^—O6—Ce1	101.7483 (5)
C6—Ce1—O6	134.183 (2)	O2^i^—O6—O5	65.5648 (11)
C6—Ce1—O16^i^	98.538 (8)	O2^i^—O6—O6^vi^	90.0184 (1)
O4^iii^—Ce1—C6	85.388 (8)	O2^i^—O6—C3	74.733 (2)
O4^iii^—Ce1—O10^ii^	121.160 (2)	O3—O6—O2^i^	110.1894 (5)
O4^iii^—Ce1—O11	106.843 (5)	O3—O6—O5^ii^	96.156 (3)
O4^iii^—Ce1—O4	103.942 (5)	O3—O6—O15	146.6079 (8)
O4^iii^—Ce1—O12	63.158 (8)	O3—O6—O15^ii^	146.6312 (8)
O4^iii^—Ce1—O17^ii^	62.9117 (10)	O3—O6—O15^iv^	146.6157 (8)
O4^iii^—Ce1—O13	64.849 (2)	O3—O6—O10^ii^	103.443 (4)
O4^iii^—Ce1—O3	45.7692 (5)	O3—O6—O17^ii^	59.102 (3)
O4^iii^—Ce1—O6	114.557 (6)	O3—O6—O16^i^	98.284 (4)
O4^iii^—Ce1—O16^i^	171.3141 (1)	O3—O6—Ce3	154.5642 (1)
C2—Ce1—O4^iii^	23.4161 (13)	O3—O6—Ce1	50.782 (8)
C2—Ce1—C6	108.784 (9)	O3—O6—O5	129.851 (8)
C2—Ce1—O10^ii^	130.042 (4)	O3—O6—O6^vi^	69.533 (3)
C2—Ce1—O11	129.519 (6)	O3—O6—C3	98.315 (8)
C2—Ce1—O4	87.318 (7)	O8^v^—O7—C4^v^	28.992 (4)
C2—Ce1—O12	85.999 (10)	O8^xx^—O7—O8^v^	57.946 (9)
C2—Ce1—O17^ii^	65.4949 (10)	O8^xx^—O7—C4^v^	28.992 (4)
C2—Ce1—O13	74.189 (4)	Ce2—O7—O8^xx^	138.8427 (11)
C2—Ce1—O3	22.3854 (18)	Ce2—O7—O8^v^	81.301 (8)
C2—Ce1—O6	96.147 (8)	Ce2—O7—C4^v^	110.229 (3)
C2—Ce1—O16^i^	151.8603 (15)	Ce2^xi^—O7—Ce2	137.900 (7)
O12^iv^—Ce2—O17	67.354 (5)	Ce2^xi^—O7—O8^xx^	81.301 (8)
O16—Ce2—O12^iv^	69.7710 (18)	Ce2^xi^—O7—O8^v^	138.8427 (11)
O16—Ce2—O17	125.5583 (9)	Ce2^xi^—O7—C4^v^	110.229 (3)
O14^i^—Ce2—O16	121.2583 (1)	C4—O7—Ce2^xi^	80.4324 (15)
O14^i^—Ce2—O12^iv^	147.535 (3)	C4—O7—Ce2	80.4324 (15)
O14^i^—Ce2—O17	112.9651 (9)	C4—O7—O8^xx^	127.7044 (19)
O14^v^—Ce2—O14^i^	0.0148 (1)	C4—O7—O8^v^	127.7044 (19)
O14^v^—Ce2—O16	121.2720 (1)	C4—O7—C4^v^	133.265
O14^v^—Ce2—O12^iv^	147.526 (3)	O8^xi^—O7—C4	26.388 (3)
O14^v^—Ce2—O17	112.9520 (9)	O8^xi^—O7—Ce2^xi^	54.7608 (15)
O14—Ce2—O14^v^	0.0288 (1)	O8^xi^—O7—Ce2	97.619 (6)
O14—Ce2—O14^i^	0.0142 (1)	O8^xi^—O7—O8^xx^	120.513 (7)
O14—Ce2—O16	121.2443 (1)	O8^xi^—O7—O8^v^	151.1679 (8)
O14—Ce2—O12^iv^	147.540 (3)	O8^xi^—O7—C4^v^	141.4088 (19)
O14—Ce2—O17	112.9788 (9)	O8—O7—O8^xi^	44.687 (7)
O2—Ce2—O14	74.0320 (1)	O8—O7—C4	26.388 (3)
O2—Ce2—O14^v^	74.0557 (1)	O8—O7—Ce2^xi^	97.619 (6)
O2—Ce2—O14^i^	74.0427 (1)	O8—O7—Ce2	54.7608 (15)
O2—Ce2—O16	67.661 (5)	O8—O7—O8^xx^	151.1679 (8)
O2—Ce2—O12^iv^	133.469 (3)	O8—O7—O8^v^	120.513 (7)
O2—Ce2—O17	127.415 (8)	O8—O7—C4^v^	141.4088 (19)
O8—Ce2—O2	147.1439 (17)	O16—O7—O8	104.073 (6)
O8—Ce2—O14	73.1120 (16)	O16—O7—O8^xi^	134.4049 (15)
O8—Ce2—O14^v^	73.0883 (16)	O16—O7—C4	130.318 (3)
O8—Ce2—O14^i^	73.1014 (16)	O16—O7—Ce2^xi^	121.223 (6)
O8—Ce2—O16	132.211 (7)	O16—O7—Ce2	52.952 (5)
O8—Ce2—O12^iv^	77.981 (5)	O16—O7—O8^xx^	100.899 (5)
O8—Ce2—O17	67.330 (5)	O16—O7—O8^v^	66.879 (4)
O1—Ce2—O8	117.556 (5)	O16—O7—C4^v^	84.1849 (4)
O1—Ce2—O2	50.941 (4)	O16^xi^—O7—O16	73.812 (10)
O1—Ce2—O14	73.1557 (16)	O16^xi^—O7—O8	134.4049 (15)
O1—Ce2—O14^v^	73.1568 (16)	O16^xi^—O7—O8^xi^	104.073 (6)
O1—Ce2—O14^i^	73.1546 (16)	O16^xi^—O7—C4	130.318 (3)
O1—Ce2—O16	110.1838 (19)	O16^xi^—O7—Ce2^xi^	52.952 (5)
O1—Ce2—O12^iv^	135.141 (2)	O16^xi^—O7—Ce2	121.223 (6)
O1—Ce2—O17	80.119 (4)	O16^xi^—O7—O8^xx^	66.879 (4)
O7—Ce2—O1	147.362 (5)	O16^xi^—O7—O8^v^	100.899 (5)
O7—Ce2—O8	68.594 (6)	O16^xi^—O7—C4^v^	84.1849 (4)
O7—Ce2—O2	105.114 (8)	O12^iv^—O7—O16^xi^	81.659 (5)
O7—Ce2—O14	79.103 (3)	O12^iv^—O7—O16	55.4402 (18)
O7—Ce2—O14^v^	79.110 (3)	O12^iv^—O7—O8	62.7510 (17)
O7—Ce2—O14^i^	79.108 (3)	O12^iv^—O7—O8^xi^	79.014 (3)
O7—Ce2—O16	70.347 (2)	O12^iv^—O7—C4	82.9107 (2)
O7—Ce2—O12^iv^	76.835 (8)	O12^iv^—O7—Ce2^xi^	90.693 (7)
O7—Ce2—O17	127.4663 (2)	O12^iv^—O7—Ce2	49.9030 (2)
O4^v^—Ce2—O7	131.117 (3)	O12^iv^—O7—O8^xx^	145.7189 (8)
O4^v^—Ce2—O1	70.685 (7)	O12^iv^—O7—O8^v^	119.059 (6)
O4^v^—Ce2—O8	132.731 (2)	O12^iv^—O7—C4^v^	139.4949 (15)
O4^v^—Ce2—O2	76.664 (5)	O12^xiii^—O7—O12^iv^	41.164 (7)
O4^v^—Ce2—O14	142.800 (5)	O12^xiii^—O7—O16^xi^	55.4402 (18)
O4^v^—Ce2—O14^v^	142.808 (5)	O12^xiii^—O7—O16	81.659 (5)
O4^v^—Ce2—O14^i^	142.802 (5)	O12^xiii^—O7—O8	79.014 (3)
O4^v^—Ce2—O16	65.488 (5)	O12^xiii^—O7—O8^xi^	62.7510 (17)
O4^v^—Ce2—O12^iv^	69.272 (8)	O12^xiii^—O7—C4	82.9107 (2)
O4^v^—Ce2—O17	68.996 (2)	O12^xiii^—O7—Ce2^xi^	49.9030 (2)
C1—Ce2—O4^v^	68.560 (6)	O12^xiii^—O7—Ce2	90.693 (7)
C1—Ce2—O7	128.720 (8)	O12^xiii^—O7—O8^xx^	119.059 (6)
C1—Ce2—O1	26.437 (4)	O12^xiii^—O7—O8^v^	145.7189 (8)
C1—Ce2—O8	139.294 (2)	O12^xiii^—O7—C4^v^	139.4949 (15)
C1—Ce2—O2	24.9380 (3)	O8^xi^—O8—C4	31.925 (5)
C1—Ce2—O14	75.2309 (8)	O7^xii^—O8—O8^xi^	61.027 (4)
C1—Ce2—O14^v^	75.2451 (8)	O7^xii^—O8—C4	29.1380 (2)
C1—Ce2—O14^i^	75.2364 (8)	Ce2—O8—O7^xii^	120.430 (3)
C1—Ce2—O16	86.399 (5)	Ce2—O8—O8^xi^	121.406 (5)
C1—Ce2—O12^iv^	137.200 (2)	Ce2—O8—C4	125.482 (4)
C1—Ce2—O17	103.417 (7)	Ce3—O8—Ce2	107.751 (7)
O15^iv^—Ce3—O17	113.0721 (11)	Ce3—O8—O7^xii^	120.217 (3)
O15^ii^—Ce3—O15^iv^	0.0288 (1)	Ce3—O8—O8^xi^	120.036 (4)
O15^ii^—Ce3—O17	113.0455 (11)	Ce3—O8—C4	126.719 (3)
O15—Ce3—O15^ii^	0.0157 (1)	O17—O8—Ce3	54.273 (3)
O15—Ce3—O15^iv^	0.0135 (1)	O17—O8—Ce2	55.206 (4)
O15—Ce3—O17	113.0607 (11)	O17—O8—O7^xii^	160.6054 (7)
O16^i^—Ce3—O15	120.8177 (1)	O17—O8—O8^xi^	138.343 (5)
O16^i^—Ce3—O15^ii^	120.8333 (1)	O17—O8—C4	170.2560 (5)
O16^i^—Ce3—O15^iv^	120.8054 (1)	O7—O8—O17	106.515 (4)
O16^i^—Ce3—O17	125.4577 (8)	O7—O8—Ce3	159.3764 (3)
O6—Ce3—O16^i^	66.315 (5)	O7—O8—Ce2	56.645 (8)
O6—Ce3—O15	74.0702 (1)	O7—O8—O7^xii^	80.402 (3)
O6—Ce3—O15^ii^	74.0817 (1)	O7—O8—O8^xi^	67.656 (4)
O6—Ce3—O15^iv^	74.0580 (1)	O7—O8—C4	70.745 (4)
O6—Ce3—O17	125.380 (8)	O16^i^—O8—O7	146.1989 (2)
O10—Ce3—O6	147.4151 (17)	O16^i^—O8—O17	101.274 (5)
O10—Ce3—O16^i^	134.128 (7)	O16^i^—O8—Ce3	53.8728 (6)
O10—Ce3—O15	73.3558 (18)	O16^i^—O8—Ce2	133.143 (6)
O10—Ce3—O15^ii^	73.3441 (18)	O16^i^—O8—O7^xii^	67.354 (4)
O10—Ce3—O15^iv^	73.3681 (18)	O16^i^—O8—O8^xi^	103.016 (2)
O10—Ce3—O17	68.368 (5)	O16^i^—O8—C4	84.711 (5)
O9—Ce3—O10	68.571 (6)	O2^i^—O8—O16^i^	56.6112 (10)
O9—Ce3—O6	107.399 (8)	O2^i^—O8—O7	124.4614 (17)
O9—Ce3—O16^i^	70.9370 (18)	O2^i^—O8—O17	59.592 (8)
O9—Ce3—O15	81.717 (3)	O2^i^—O8—Ce3	55.889 (4)
O9—Ce3—O15^ii^	81.723 (3)	O2^i^—O8—Ce2	77.180 (8)
O9—Ce3—O15^iv^	81.716 (3)	O2^i^—O8—O7^xii^	101.386 (7)
O9—Ce3—O17	127.1439 (1)	O2^i^—O8—O8^xi^	158.555 (3)
O8—Ce3—O9	76.944 (7)	O2^i^—O8—C4	129.877 (7)
O8—Ce3—O10	80.589 (4)	O14^i^—O8—O2^i^	60.866 (4)
O8—Ce3—O6	131.189 (3)	O14^i^—O8—O16^i^	94.132 (3)
O8—Ce3—O16^i^	70.0316 (16)	O14^i^—O8—O7	66.678 (6)
O8—Ce3—O15	150.962 (3)	O14^i^—O8—O17	91.380 (8)
O8—Ce3—O15^ii^	150.957 (3)	O14^i^—O8—Ce3	116.652 (8)
O8—Ce3—O15^iv^	150.971 (3)	O14^i^—O8—Ce2	52.568 (3)
O8—Ce3—O17	67.126 (5)	O14^i^—O8—O7^xii^	74.491 (6)
O2^i^—Ce3—O8	68.994 (8)	O14^i^—O8—O8^xi^	119.792 (4)
O2^i^—Ce3—O9	131.611 (3)	O14^i^—O8—C4	95.883 (8)
O2^i^—Ce3—O10	134.069 (2)	O14^v^—O8—O14^i^	0.0237 (1)
O2^i^—Ce3—O6	73.967 (5)	O14^v^—O8—O2^i^	60.858 (4)
O2^i^—Ce3—O16^i^	65.755 (5)	O14^v^—O8—O16^i^	94.110 (3)
O2^i^—Ce3—O15	139.575 (5)	O14^v^—O8—O7	66.693 (6)
O2^i^—Ce3—O15^ii^	139.577 (5)	O14^v^—O8—O17	91.393 (8)
O2^i^—Ce3—O15^iv^	139.569 (5)	O14^v^—O8—Ce3	116.646 (8)
O2^i^—Ce3—O17	68.407 (2)	O14^v^—O8—Ce2	52.591 (3)
O5—Ce3—O2^i^	69.431 (7)	O14^v^—O8—O7^xii^	74.474 (6)
O5—Ce3—O8	132.955 (2)	O14^v^—O8—O8^xi^	119.790 (4)
O5—Ce3—O9	149.705 (5)	O14^v^—O8—C4	95.872 (8)
O5—Ce3—O10	115.072 (5)	O14—O8—O14^v^	0.0176 (1)
O5—Ce3—O6	52.110 (4)	O14—O8—O14^i^	0.0141 (1)
O5—Ce3—O16^i^	110.7371 (15)	O14—O8—O2^i^	60.873 (4)
O5—Ce3—O15	71.5659 (14)	O14—O8—O16^i^	94.127 (3)
O5—Ce3—O15^ii^	71.5639 (14)	O14—O8—O7	66.676 (6)
O5—Ce3—O15^iv^	71.5636 (14)	O14—O8—O17	91.394 (8)
O5—Ce3—O17	77.882 (4)	O14—O8—Ce3	116.660 (8)
C3—Ce3—O5	27.422 (4)	O14—O8—Ce2	52.579 (3)
C3—Ce3—O2^i^	65.410 (6)	O14—O8—O7^xii^	74.477 (6)
C3—Ce3—O8	133.911 (2)	O14—O8—O8^xi^	119.780 (4)
C3—Ce3—O9	131.591 (7)	O14—O8—C4	95.869 (8)
C3—Ce3—O10	138.6430 (19)	O12^iv^—O8—O14	101.5289 (5)
C3—Ce3—O6	25.3362 (4)	O12^iv^—O8—O14^v^	101.5426 (5)
C3—Ce3—O16^i^	85.402 (5)	O12^iv^—O8—O14^i^	101.5189 (5)
C3—Ce3—O15	75.1264 (4)	O12^iv^—O8—O2^i^	111.008 (3)
C3—Ce3—O15^ii^	75.1320 (4)	O12^iv^—O8—O16^i^	151.0849 (10)
C3—Ce3—O15^iv^	75.1180 (4)	O12^iv^—O8—O7	62.6684 (8)
C3—Ce3—O17	101.092 (8)	O12^iv^—O8—O17	54.654 (4)
O10^ii^—Ce3—C3	84.690 (8)	O12^iv^—O8—Ce3	97.2380 (4)
O10^ii^—Ce3—O5	106.178 (5)	O12^iv^—O8—Ce2	50.158 (2)
O10^ii^—Ce3—O2^i^	119.289 (3)	O12^iv^—O8—O7^xii^	140.302 (3)
O10^ii^—Ce3—O8	112.721 (6)	O12^iv^—O8—O8^xi^	90.1610 (1)
O10^ii^—Ce3—O9	46.9082 (7)	O12^iv^—O8—C4	117.207 (4)
O10^ii^—Ce3—O10	103.689 (5)	O9—O8—O12^iv^	105.7773 (3)
O10^ii^—Ce3—O6	61.182 (8)	O9—O8—O14	151.1353 (5)
O10^ii^—Ce3—O16^i^	60.0063 (11)	O9—O8—O14^v^	151.1187 (5)
O10^ii^—Ce3—O15	62.8915 (17)	O9—O8—O14^i^	151.1414 (5)
O10^ii^—Ce3—O15^ii^	62.9060 (17)	O9—O8—O2^i^	99.973 (5)
O10^ii^—Ce3—O15^iv^	62.8812 (17)	O9—O8—O16^i^	57.150 (3)
O10^ii^—Ce3—O17	172.0527 (4)	O9—O8—O7	135.531 (7)
O2—C1—O2^vi^	124.551 (8)	O9—O8—O17	96.757 (5)
O1—C1—O2	117.717 (4)	O9—O8—Ce3	50.931 (8)
O1—C1—O2^vi^	117.717 (4)	O9—O8—Ce2	149.4959 (5)
O1^v^—C1—O1	93.782	O9—O8—O7^xii^	90.021 (3)
O1^v^—C1—O2	87.6638 (3)	O9—O8—O8^xi^	69.739 (3)
O1^v^—C1—O2^vi^	87.6638 (3)	O9—O8—C4	79.930 (5)
Ce2—C1—O1^v^	103.3057 (18)	O10^xvii^—O9—C5^ii^	28.379 (4)
Ce2—C1—O1	61.442 (4)	O10^ii^—O9—O10^xvii^	56.759 (9)
Ce2—C1—O2	57.797 (9)	O10^ii^—O9—C5^ii^	28.379 (4)
Ce2—C1—O2^vi^	168.9995 (14)	Ce3—O9—O10^ii^	80.410 (7)
Ce2^vi^—C1—Ce2	117.627 (9)	Ce3—O9—O10^xvii^	136.9046 (12)
Ce2^vi^—C1—O1^v^	103.3057 (18)	Ce3—O9—C5^ii^	108.687 (3)
Ce2^vi^—C1—O1	61.442 (4)	Ce3^xi^—O9—Ce3	141.262 (6)
Ce2^vi^—C1—O2	168.9995 (14)	Ce3^xi^—O9—O10^ii^	136.9046 (12)
Ce2^vi^—C1—O2^vi^	57.797 (9)	Ce3^xi^—O9—O10^xvii^	80.410 (7)
O4^vii^—C1—Ce2^vi^	51.6872 (9)	Ce3^xi^—O9—C5^ii^	108.687 (3)
O4^vii^—C1—Ce2	88.742 (6)	C5—O9—Ce3^xi^	81.5567 (13)
O4^vii^—C1—O1^v^	154.808 (3)	C5—O9—Ce3	81.5567 (13)
O4^vii^—C1—O1	72.4156 (4)	C5—O9—O10^ii^	125.5038 (16)
O4^vii^—C1—O2	117.314 (2)	C5—O9—O10^xvii^	125.5038 (16)
O4^vii^—C1—O2^vi^	80.799 (5)	C5—O9—C5^ii^	131.242
O4^v^—C1—O4^vii^	40.914 (7)	O10—O9—C5	26.913 (3)
O4^v^—C1—Ce2^vi^	88.742 (6)	O10—O9—Ce3^xi^	99.576 (6)
O4^v^—C1—Ce2	51.6872 (9)	O10—O9—Ce3	55.4960 (18)
O4^v^—C1—O1^v^	154.808 (3)	O10—O9—O10^ii^	119.135 (6)
O4^v^—C1—O1	72.4156 (4)	O10—O9—O10^xvii^	148.8802 (9)
O4^v^—C1—O2	80.799 (5)	O10—O9—C5^ii^	139.6961 (18)
O4^v^—C1—O2^vi^	117.314 (2)	O10^xi^—O9—O10	45.709 (7)
O4^viii^—C2—O4^iii^	121.864 (9)	O10^xi^—O9—C5	26.913 (3)
O3—C2—O4^viii^	119.033 (4)	O10^xi^—O9—Ce3^xi^	55.4960 (18)
O3—C2—O4^iii^	119.033 (4)	O10^xi^—O9—Ce3	99.576 (6)
O3^viii^—C2—O3	107.518	O10^xi^—O9—O10^ii^	148.8802 (9)
O3^viii^—C2—O4^viii^	82.8618 (10)	O10^xi^—O9—O10^xvii^	119.135 (6)
O3^viii^—C2—O4^iii^	82.8618 (10)	O10^xi^—O9—C5^ii^	139.6961 (18)
O17^ix^—C2—O3^viii^	145.484 (5)	O16^xii^—O9—O10^xi^	106.661 (7)
O17^ix^—C2—O3	71.8604 (11)	O16^xii^—O9—O10	138.9005 (13)
O17^ix^—C2—O4^viii^	69.006 (7)	O16^xii^—O9—C5	133.546 (4)
O17^ix^—C2—O4^iii^	128.713 (4)	O16^xii^—O9—Ce3^xi^	54.104 (5)
O17^ii^—C2—O17^ix^	68.489 (10)	O16^xii^—O9—Ce3	123.206 (5)
O17^ii^—C2—O3^viii^	145.484 (5)	O16^xii^—O9—O10^ii^	98.820 (5)
O17^ii^—C2—O3	71.8604 (11)	O16^xii^—O9—O10^xvii^	65.293 (4)
O17^ii^—C2—O4^viii^	128.713 (4)	O16^xii^—O9—C5^ii^	81.4009 (6)
O17^ii^—C2—O4^iii^	69.006 (7)	O16^i^—O9—O16^xii^	73.883 (10)
O1^ii^—C2—O17^ii^	62.5502 (17)	O16^i^—O9—O10^xi^	138.9005 (13)
O1^ii^—C2—O17^ix^	62.5502 (17)	O16^i^—O9—O10	106.661 (7)
O1^ii^—C2—O3^viii^	128.500	O16^i^—O9—C5	133.546 (4)
O1^ii^—C2—O3	123.982	O16^i^—O9—Ce3^xi^	123.206 (5)
O1^ii^—C2—O4^viii^	73.128 (2)	O16^i^—O9—Ce3	54.104 (5)
O1^ii^—C2—O4^iii^	73.128 (2)	O16^i^—O9—O10^ii^	65.293 (4)
Ce1^vi^—C2—O1^ii^	108.2480 (18)	O16^i^—O9—O10^xvii^	98.820 (5)
Ce1^vi^—C2—O17^ii^	98.211 (7)	O16^i^—O9—C5^ii^	81.4009 (6)
Ce1^vi^—C2—O17^ix^	46.966 (3)	O8—O9—O16^i^	56.9140 (19)
Ce1^vi^—C2—O3^viii^	106.1066 (16)	O8—O9—O16^xii^	82.551 (5)
Ce1^vi^—C2—O3	47.507 (5)	O8—O9—O10^xi^	82.133 (3)
Ce1^vi^—C2—O4^viii^	71.648 (9)	O8—O9—O10	66.0540 (18)
Ce1^vi^—C2—O4^iii^	165.2278 (9)	O8—O9—C5	86.5430 (1)
Ce1—C2—Ce1^vi^	94.321 (10)	O8—O9—Ce3^xi^	92.261 (6)
Ce1—C2—O1^ii^	108.2480 (18)	O8—O9—Ce3	52.1246 (4)
Ce1—C2—O17^ii^	46.966 (3)	O8—O9—O10^ii^	119.285 (6)
Ce1—C2—O17^ix^	98.211 (7)	O8—O9—O10^xvii^	144.9201 (8)
Ce1—C2—O3^viii^	106.1066 (16)	O8—O9—C5^ii^	138.0472 (14)
Ce1—C2—O3	47.507 (5)	O8^xi^—O9—O8	40.522 (7)
Ce1—C2—O4^viii^	165.2278 (9)	O8^xi^—O9—O16^i^	82.551 (5)
Ce1—C2—O4^iii^	71.648 (9)	O8^xi^—O9—O16^xii^	56.9140 (19)
O6^vi^—C3—O6	121.458 (9)	O8^xi^—O9—O10^xi^	66.0540 (18)
O5—C3—O6^vi^	119.079 (4)	O8^xi^—O9—O10	82.133 (3)
O5—C3—O6	119.079 (4)	O8^xi^—O9—C5	86.5430 (1)
O5^ii^—C3—O5	91.626	O8^xi^—O9—Ce3^xi^	52.1246 (4)
O5^ii^—C3—O6^vi^	86.1468 (5)	O8^xi^—O9—Ce3	92.261 (6)
O5^ii^—C3—O6	86.1468 (5)	O8^xi^—O9—O10^ii^	144.9201 (8)
Ce3^vi^—C3—O5^ii^	102.6532 (17)	O8^xi^—O9—O10^xvii^	119.285 (6)
Ce3^vi^—C3—O5	62.937 (4)	O8^xi^—O9—C5^ii^	138.0472 (14)
Ce3^vi^—C3—O6^vi^	58.429 (9)	O10^xi^—O10—C5	32.265 (5)
Ce3^vi^—C3—O6	171.0512 (12)	O9^xiii^—O10—O10^xi^	61.621 (4)
Ce3—C3—Ce3^vi^	120.063 (9)	O9^xiii^—O10—C5	29.3553 (3)
Ce3—C3—O5^ii^	102.6532 (17)	Ce3—O10—O9^xiii^	120.993 (3)
Ce3—C3—O5	62.937 (4)	Ce3—O10—O10^xi^	120.637 (4)
Ce3—C3—O6^vi^	171.0512 (12)	Ce3—O10—C5	126.545 (4)
Ce3—C3—O6	58.429 (9)	Ce1^iv^—O10—Ce3	106.020 (8)
O2^x^—C3—Ce3	91.113 (6)	Ce1^iv^—O10—O9^xiii^	119.025 (3)
O2^x^—C3—Ce3^vi^	51.9188 (12)	Ce1^iv^—O10—O10^xi^	123.673 (5)
O2^x^—C3—O5^ii^	154.494 (3)	Ce1^iv^—O10—C5	127.175 (4)
O2^x^—C3—O5	75.4961 (4)	O17—O10—Ce1^iv^	54.049 (4)
O2^x^—C3—O6^vi^	81.331 (5)	O17—O10—Ce3	54.618 (4)
O2^x^—C3—O6	119.334 (2)	O17—O10—O9^xiii^	160.4711 (8)
O2^i^—C3—O2^x^	43.086 (7)	O17—O10—O10^xi^	137.900 (5)
O2^i^—C3—Ce3	51.9188 (12)	O17—O10—C5	170.1587 (5)
O2^i^—C3—Ce3^vi^	91.113 (6)	O9—O10—O17	104.600 (4)
O2^i^—C3—O5^ii^	154.494 (3)	O9—O10—Ce1^iv^	158.1028 (1)
O2^i^—C3—O5	75.4961 (4)	O9—O10—Ce3	55.933 (8)
O2^i^—C3—O6^vi^	119.334 (2)	O9—O10—O9^xiii^	82.711 (3)
O2^i^—C3—O6	81.331 (5)	O9—O10—O10^xi^	67.145 (4)
O8^xi^—C4—O8	116.151 (9)	O9—O10—C5	72.875 (4)
O7^xii^—C4—O8^xi^	121.870 (5)	O16^xiv^—O10—O9	149.1032 (1)
O7^xii^—C4—O8	121.870 (5)	O16^xiv^—O10—O17	101.503 (5)
O7—C4—O7^xii^	106.735	O16^xiv^—O10—Ce1^iv^	51.8420 (4)
O7—C4—O8^xi^	82.8679 (9)	O16^xiv^—O10—Ce3	134.274 (6)
O7—C4—O8	82.8679 (9)	O16^xiv^—O10—O9^xiii^	67.438 (4)
O16^xii^—C4—O7	144.656 (5)	O16^xiv^—O10—O10^xi^	103.098 (2)
O16^xii^—C4—O7^xii^	70.9352 (11)	O16^xiv^—O10—C5	83.937 (5)
O16^xii^—C4—O8^xi^	70.508 (7)	O6^iv^—O10—O16^xiv^	56.4598 (10)
O16^xii^—C4—O8	129.400 (4)	O6^iv^—O10—O9	126.0291 (16)
O16^i^—C4—O16^xii^	69.570 (10)	O6^iv^—O10—O17	60.083 (8)
O16^i^—C4—O7	144.656 (5)	O6^iv^—O10—Ce1^iv^	51.498 (4)
O16^i^—C4—O7^xii^	70.9352 (11)	O6^iv^—O10—Ce3	78.283 (8)
O16^i^—C4—O8^xi^	129.400 (4)	O6^iv^—O10—O9^xiii^	100.852 (7)
O16^i^—C4—O8	70.508 (7)	O6^iv^—O10—O10^xi^	158.488 (3)
O10—C5—O10^xi^	115.469 (9)	O6^iv^—O10—C5	129.262 (8)
O9^xiii^—C5—O10	122.265 (5)	O15^ii^—O10—O6^iv^	61.104 (5)
O9^xiii^—C5—O10^xi^	122.265 (5)	O15^ii^—O10—O16^xiv^	95.315 (3)
O9—C5—O9^xiii^	108.758	O15^ii^—O10—O9	68.361 (6)
O9—C5—O10	80.2118 (13)	O15^ii^—O10—O17	90.609 (8)
O9—C5—O10^xi^	80.2118 (13)	O15^ii^—O10—Ce1^iv^	112.460 (9)
O16^xiv^—C5—O9	144.467 (5)	O15^ii^—O10—Ce3	52.658 (3)
O16^xiv^—C5—O9^xiii^	72.5069 (11)	O15^ii^—O10—O9^xiii^	75.080 (6)
O16^xiv^—C5—O10	70.667 (7)	O15^ii^—O10—O10^xi^	120.220 (4)
O16^xiv^—C5—O10^xi^	130.492 (4)	O15^ii^—O10—C5	97.092 (8)
O16^xv^—C5—O16^xiv^	70.862 (10)	O11^xiii^—O10—O15^ii^	159.6112 (2)
O16^xv^—C5—O9	144.467 (5)	O11^xiii^—O10—O6^iv^	103.857 (5)
O16^xv^—C5—O9^xiii^	72.5069 (11)	O11^xiii^—O10—O16^xiv^	64.301 (3)
O16^xv^—C5—O10	130.492 (4)	O11^xiii^—O10—O9	129.560 (6)
O16^xv^—C5—O10^xi^	70.667 (7)	O11^xiii^—O10—O17	93.051 (5)
O11^xiii^—C5—O16^xv^	61.3519 (19)	O11^xiii^—O10—Ce1^iv^	55.465 (8)
O11^xiii^—C5—O16^xiv^	61.3519 (19)	O11^xiii^—O10—Ce3	142.1697 (11)
O11^xiii^—C5—O9	128.934	O11^xiii^—O10—O9^xiii^	96.025 (4)
O11^xiii^—C5—O9^xiii^	122.309	O11^xiii^—O10—O10^xi^	68.259 (4)
O11^xiii^—C5—O10	73.333 (2)	O11^xiii^—O10—C5	81.854 (5)
O11^xiii^—C5—O10^xi^	73.333 (2)	O15^iv^—O10—O11^xiii^	159.6293 (2)
O12—C6—O12^xi^	122.755 (9)	O15^iv^—O10—O15^ii^	0.0183 (1)
O11—C6—O12	118.579 (4)	O15^iv^—O10—O6^iv^	61.119 (5)
O11—C6—O12^xi^	118.579 (4)	O15^iv^—O10—O16^xiv^	95.333 (3)
O11^xvi^—C6—O11	94.940	O15^iv^—O10—O9	68.343 (6)
O11^xvi^—C6—O12	89.0925 (1)	O15^iv^—O10—O17	90.608 (8)
O11^xvi^—C6—O12^xi^	89.0925 (1)	O15^iv^—O10—Ce1^iv^	112.474 (9)
Ce1—C6—O11^xvi^	102.3527 (17)	O15^iv^—O10—Ce3	52.646 (3)
Ce1—C6—O11	63.163 (4)	O15^iv^—O10—O9^xiii^	75.084 (6)
Ce1—C6—O12	56.191 (8)	O15^iv^—O10—O10^xi^	120.211 (4)
Ce1—C6—O12^xi^	168.3507 (16)	O15^iv^—O10—C5	97.090 (8)
Ce1^xi^—C6—Ce1	122.047 (9)	O15—O10—O15^iv^	0.0236 (1)
Ce1^xi^—C6—O11^xvi^	102.3527 (17)	O15—O10—O11^xiii^	159.6080 (2)
Ce1^xi^—C6—O11	63.163 (4)	O15—O10—O15^ii^	0.0137 (1)
Ce1^xi^—C6—O12	168.3507 (16)	O15—O10—O6^iv^	61.112 (5)
Ce1^xi^—C6—O12^xi^	56.191 (8)	O15—O10—O16^xiv^	95.311 (3)
O10^ii^—C6—Ce1^xi^	91.202 (6)	O15—O10—O9	68.358 (6)
O10^ii^—C6—Ce1	52.9355 (11)	O15—O10—O17	90.622 (8)
O10^ii^—C6—O11^xvi^	155.149 (3)	O15—O10—Ce1^iv^	112.468 (9)
O10^ii^—C6—O11	72.5484 (4)	O15—O10—Ce3	52.669 (3)
O10^ii^—C6—O12	78.933 (5)	O15—O10—O9^xiii^	75.066 (6)
O10^ii^—C6—O12^xi^	115.712 (3)	O15—O10—O10^xi^	120.208 (4)
O10^xvii^—C6—O10^ii^	41.766 (7)	O15—O10—C5	97.078 (8)
O10^xvii^—C6—Ce1^xi^	52.9355 (11)	C6^xiii^—O10—O15	169.1399 (8)
O10^xvii^—C6—Ce1	91.202 (6)	C6^xiii^—O10—O15^iv^	169.1253 (8)
O10^xvii^—C6—O11^xvi^	155.149 (3)	C6^xiii^—O10—O11^xiii^	25.0167 (6)
O10^xvii^—C6—O11	72.5484 (4)	C6^xiii^—O10—O15^ii^	169.1263 (8)
O10^xvii^—C6—O12	115.712 (3)	C6^xiii^—O10—O6^iv^	112.620 (3)
O10^xvii^—C6—O12^xi^	78.933 (5)	C6^xiii^—O10—O16^xiv^	87.3475 (17)
O2^vi^—O1—C1	30.327 (4)	C6^xiii^—O10—O9	113.740 (4)
O2—O1—O2^vi^	60.651 (9)	C6^xiii^—O10—O17	78.525 (7)
O2—O1—C1	30.327 (4)	C6^xiii^—O10—Ce1^iv^	61.314 (7)
Ce2^vi^—O1—O2	121.647 (4)	C6^xiii^—O10—Ce3	118.783 (2)
Ce2^vi^—O1—O2^vi^	62.730 (5)	C6^xiii^—O10—O9^xiii^	115.569 (6)
Ce2^vi^—O1—C1	92.1206 (4)	C6^xiii^—O10—O10^xi^	69.117 (3)
Ce2—O1—Ce2^vi^	153.468 (5)	C6^xiii^—O10—C5	93.669 (7)
Ce2—O1—O2	62.730 (5)	O12^iv^—O10—C6^xiii^	23.456 (3)
Ce2—O1—O2^vi^	121.647 (4)	O12^iv^—O10—O15	145.687 (4)
Ce2—O1—C1	92.1206 (4)	O12^iv^—O10—O15^iv^	145.672 (4)
C1^x^—O1—Ce2	95.4609 (9)	O12^iv^—O10—O11^xiii^	42.6975 (15)
C1^x^—O1—Ce2^vi^	95.4609 (9)	O12^iv^—O10—O15^ii^	145.673 (4)
C1^x^—O1—O2	135.579 (3)	O12^iv^—O10—O6^iv^	97.0249 (11)
C1^x^—O1—O2^vi^	135.579 (3)	O12^iv^—O10—O16^xiv^	92.6673 (1)
C1^x^—O1—C1	146.218	O12^iv^—O10—O9	115.8011 (7)
O14^i^—O1—C1^x^	67.955 (3)	O12^iv^—O10—O17	55.071 (4)
O14^i^—O1—Ce2	52.001 (7)	O12^iv^—O10—Ce1^iv^	50.441 (3)
O14^i^—O1—Ce2^vi^	153.874 (3)	O12^iv^—O10—Ce3	99.5132 (5)
O14^i^—O1—O2	68.340 (5)	O12^iv^—O10—O9^xiii^	138.143 (3)
O14^i^—O1—O2^vi^	114.584 (5)	O12^iv^—O10—O10^xi^	90.0725 (1)
O14^i^—O1—C1	91.7983 (2)	O12^iv^—O10—C5	116.952 (4)
O14^x^—O1—O14^i^	102.062 (10)	Ce3^iv^—O10—O12^iv^	137.0700 (19)
O14^x^—O1—C1^x^	67.955 (3)	Ce3^iv^—O10—C6^xiii^	136.3159 (12)
O14^x^—O1—Ce2	153.874 (3)	Ce3^iv^—O10—O15	47.6063 (18)
O14^x^—O1—Ce2^vi^	52.001 (7)	Ce3^iv^—O10—O15^iv^	47.6294 (18)
O14^x^—O1—O2	114.584 (5)	Ce3^iv^—O10—O11^xiii^	112.434 (2)
O14^x^—O1—O2^vi^	68.340 (5)	Ce3^iv^—O10—O15^ii^	47.6125 (18)
O14^x^—O1—C1	91.7983 (2)	Ce3^iv^—O10—O6^iv^	48.7979 (11)
O14—O1—O14^x^	102.046 (10)	Ce3^iv^—O10—O16^xiv^	48.9878 (6)
O14—O1—O14^i^	0.0201 (1)	Ce3^iv^—O10—O9	106.396 (2)
O14—O1—C1^x^	67.958 (2)	Ce3^iv^—O10—O17	107.820 (9)
O14—O1—Ce2	52.017 (7)	Ce3^iv^—O10—Ce1^iv^	86.992 (4)
O14—O1—Ce2^vi^	153.860 (3)	Ce3^iv^—O10—Ce3	97.432 (5)
O14—O1—O2	68.334 (5)	Ce3^iv^—O10—O9^xiii^	52.682 (8)
O14—O1—O2^vi^	114.567 (5)	Ce3^iv^—O10—O10^xi^	114.135 (4)
O14—O1—C1	91.7863 (2)	Ce3^iv^—O10—C5	81.956 (8)
O14^vi^—O1—O14	102.030 (10)	O12^xi^—O11—C6	29.933 (4)
O14^vi^—O1—O14^x^	0.0201 (1)	O12—O11—O12^xi^	59.839 (9)
O14^vi^—O1—O14^i^	102.046 (10)	O12—O11—C6	29.933 (4)
O14^vi^—O1—C1^x^	67.958 (2)	Ce1—O11—O12	61.054 (5)
O14^vi^—O1—Ce2	153.860 (3)	Ce1—O11—O12^xi^	119.604 (4)
O14^vi^—O1—Ce2^vi^	52.017 (7)	Ce1—O11—C6	90.5190 (1)
O14^vi^—O1—O2	114.567 (5)	Ce1^xi^—O11—Ce1	157.263 (4)
O14^vi^—O1—O2^vi^	68.334 (5)	Ce1^xi^—O11—O12	119.604 (4)
O14^vi^—O1—C1	91.7863 (2)	Ce1^xi^—O11—O12^xi^	61.054 (5)
O14^v^—O1—O14^vi^	102.030 (10)	Ce1^xi^—O11—C6	90.5190 (1)
O14^v^—O1—O14	0.0237 (1)	O13^xvi^—O11—Ce1^xi^	52.168 (7)
O14^v^—O1—O14^x^	102.045 (10)	O13^xvi^—O11—Ce1	150.491 (3)
O14^v^—O1—O14^i^	0.0197 (1)	O13^xvi^—O11—O12	113.974 (5)
O14^v^—O1—C1^x^	67.937 (3)	O13^xvi^—O11—O12^xi^	69.605 (5)
O14^v^—O1—Ce2	52.016 (7)	O13^xvi^—O11—C6	91.2868 (1)
O14^v^—O1—Ce2^vi^	153.856 (3)	O13—O11—O13^xvi^	98.341 (10)
O14^v^—O1—O2	68.357 (5)	O13—O11—Ce1^xi^	150.491 (3)
O14^v^—O1—O2^vi^	114.589 (5)	O13—O11—Ce1	52.168 (7)
O14^v^—O1—C1	91.8100 (2)	O13—O11—O12	69.605 (5)
O14^vii^—O1—O14^v^	102.029 (10)	O13—O11—O12^xi^	113.974 (5)
O14^vii^—O1—O14^vi^	0.0237 (1)	O13—O11—C6	91.2868 (1)
O14^vii^—O1—O14	102.030 (10)	C6^xix^—O11—O13	66.882 (2)
O14^vii^—O1—O14^x^	0.0197 (1)	C6^xix^—O11—O13^xvi^	66.882 (2)
O14^vii^—O1—O14^i^	102.045 (10)	C6^xix^—O11—Ce1^xi^	96.0472 (10)
O14^vii^—O1—C1^x^	67.937 (3)	C6^xix^—O11—Ce1	96.0472 (10)
O14^vii^—O1—Ce2	153.856 (3)	C6^xix^—O11—O12	135.946 (3)
O14^vii^—O1—Ce2^vi^	52.016 (7)	C6^xix^—O11—O12^xi^	135.946 (3)
O14^vii^—O1—O2	114.589 (5)	C6^xix^—O11—C6	145.060
O14^vii^—O1—O2^vi^	68.357 (5)	O10^ii^—O11—C6^xix^	129.3850 (12)
O14^vii^—O1—C1	91.8100 (2)	O10^ii^—O11—O13	108.875 (9)
O4^vii^—O1—O14^vii^	107.531 (8)	O10^ii^—O11—O13^xvi^	152.1346 (15)
O4^vii^—O1—O14^v^	150.1528 (15)	O10^ii^—O11—Ce1^xi^	100.561 (5)
O4^vii^—O1—O14^vi^	107.529 (8)	O10^ii^—O11—Ce1	57.1114 (16)
O4^vii^—O1—O14	150.1488 (15)	O10^ii^—O11—O12	71.812 (3)
O4^vii^—O1—O14^x^	107.514 (8)	O10^ii^—O11—O12^xi^	93.289 (4)
O4^vii^—O1—O14^i^	150.1351 (15)	O10^ii^—O11—C6	82.4348 (2)
O4^vii^—O1—C1^x^	127.6082 (10)	O10^xvii^—O11—O10^ii^	43.482 (7)
O4^vii^—O1—Ce2	98.596 (6)	O10^xvii^—O11—C6^xix^	129.3850 (12)
O4^vii^—O1—Ce2^vi^	55.9841 (13)	O10^xvii^—O11—O13	152.1346 (15)
O4^vii^—O1—O2	95.108 (3)	O10^xvii^—O11—O13^xvi^	108.875 (9)
O4^vii^—O1—O2^vi^	73.818 (3)	O10^xvii^—O11—Ce1^xi^	57.1114 (16)
O4^vii^—O1—C1	83.3367 (2)	O10^xvii^—O11—Ce1	100.561 (5)
O4^v^—O1—O4^vii^	42.712 (7)	O10^xvii^—O11—O12	93.289 (4)
O4^v^—O1—O14^vii^	150.1528 (15)	O10^xvii^—O11—O12^xi^	71.812 (3)
O4^v^—O1—O14^v^	107.531 (8)	O10^xvii^—O11—C6	82.4348 (2)
O4^v^—O1—O14^vi^	150.1488 (15)	C5^ii^—O11—O10^xvii^	24.812 (3)
O4^v^—O1—O14	107.529 (8)	C5^ii^—O11—O10^ii^	24.812 (3)
O4^v^—O1—O14^x^	150.1351 (15)	C5^ii^—O11—C6^xix^	120.828
O4^v^—O1—O14^i^	107.514 (8)	C5^ii^—O11—O13	130.552 (5)
O4^v^—O1—C1^x^	127.6082 (10)	C5^ii^—O11—O13^xvi^	130.552 (5)
O4^v^—O1—Ce2	55.9841 (13)	C5^ii^—O11—Ce1^xi^	78.635 (2)
O4^v^—O1—Ce2^vi^	98.596 (6)	C5^ii^—O11—Ce1	78.635 (2)
O4^v^—O1—O2	73.818 (3)	C5^ii^—O11—O12	92.7345 (1)
O4^v^—O1—O2^vi^	95.108 (3)	C5^ii^—O11—O12^xi^	92.7345 (1)
O4^v^—O1—C1	83.3367 (2)	C5^ii^—O11—C6	94.111
O2^i^—O1—O4^v^	107.621 (4)	O16^i^—O11—C5^ii^	58.493 (2)
O2^i^—O1—O4^vii^	123.9665 (14)	O16^i^—O11—O10^xvii^	81.981 (5)
O2^i^—O1—O14^vii^	91.220 (5)	O16^i^—O11—O10^ii^	56.170 (2)
O2^i^—O1—O14^v^	58.322 (2)	O16^i^—O11—C6^xix^	73.6314 (10)
O2^i^—O1—O14^vi^	91.240 (5)	O16^i^—O11—O13	82.489 (8)
O2^i^—O1—O14	58.346 (2)	O16^i^—O11—O13^xvi^	136.186 (3)
O2^i^—O1—O14^x^	91.238 (5)	O16^i^—O11—Ce1^xi^	116.900 (6)
O2^i^—O1—O14^i^	58.335 (2)	O16^i^—O11—Ce1	49.316 (4)
O2^i^—O1—C1^x^	23.375 (3)	O16^i^—O11—O12	107.334 (8)
O2^i^—O1—Ce2	72.952 (4)	O16^i^—O11—O12^xi^	149.2911 (15)
O2^i^—O1—Ce2^vi^	113.019 (3)	O16^i^—O11—C6	132.523 (3)
O2^i^—O1—O2	124.564 (7)	O16^xii^—O11—O16^i^	68.556 (10)
O2^i^—O1—O2^vi^	157.2598 (2)	O16^xii^—O11—C5^ii^	58.493 (2)
O2^i^—O1—C1	149.913 (2)	O16^xii^—O11—O10^xvii^	56.170 (2)
O2^x^—O1—O2^i^	41.157 (7)	O16^xii^—O11—O10^ii^	81.981 (5)
O2^x^—O1—O4^v^	123.9665 (14)	O16^xii^—O11—C6^xix^	73.6314 (10)
O2^x^—O1—O4^vii^	107.621 (4)	O16^xii^—O11—O13	136.186 (3)
O2^x^—O1—O14^vii^	58.322 (2)	O16^xii^—O11—O13^xvi^	82.489 (8)
O2^x^—O1—O14^v^	91.220 (5)	O16^xii^—O11—Ce1^xi^	49.316 (4)
O2^x^—O1—O14^vi^	58.346 (2)	O16^xii^—O11—Ce1	116.900 (6)
O2^x^—O1—O14	91.240 (5)	O16^xii^—O11—O12	149.2911 (15)
O2^x^—O1—O14^x^	58.335 (2)	O16^xii^—O11—O12^xi^	107.334 (8)
O2^x^—O1—O14^i^	91.238 (5)	O16^xii^—O11—C6	132.523 (3)
O2^x^—O1—C1^x^	23.375 (3)	O12^xi^—O12—C6	28.623 (4)
O2^x^—O1—Ce2	113.019 (3)	O11—O12—O12^xi^	60.081 (4)
O2^x^—O1—Ce2^vi^	72.952 (4)	O11—O12—C6	31.4882 (1)
O2^x^—O1—O2	157.2598 (2)	Ce2^ii^—O12—O11	169.4311 (4)
O2^x^—O1—O2^vi^	124.564 (7)	Ce2^ii^—O12—O12^xi^	122.020 (5)
O2^x^—O1—C1	149.913 (2)	Ce2^ii^—O12—C6	149.9301 (4)
C2^xviii^—O1—O2^x^	108.2704 (4)	Ce1—O12—Ce2^ii^	106.713 (9)
C2^xviii^—O1—O2^i^	108.2704 (4)	Ce1—O12—O11	68.122 (9)
C2^xviii^—O1—O4^v^	23.497 (3)	Ce1—O12—O12^xi^	126.712 (5)
C2^xviii^—O1—O4^vii^	23.497 (3)	Ce1—O12—C6	99.084 (9)
C2^xviii^—O1—O14^vii^	128.752 (5)	O17^ii^—O12—Ce1	56.648 (5)
C2^xviii^—O1—O14^v^	128.752 (5)	O17^ii^—O12—Ce2^ii^	56.230 (4)
C2^xviii^—O1—O14^vi^	128.754 (5)	O17^ii^—O12—O11	114.676 (5)
C2^xviii^—O1—O14	128.754 (5)	O17^ii^—O12—O12^xi^	138.845 (5)
C2^xviii^—O1—O14^x^	128.737 (5)	O17^ii^—O12—C6	134.071 (6)
C2^xviii^—O1—O14^i^	128.737 (5)	O16^ii^—O12—O17^ii^	104.185 (5)
C2^xviii^—O1—C1^x^	120.900	O16^ii^—O12—Ce1	122.770 (6)
C2^xviii^—O1—Ce2	76.818 (2)	O16^ii^—O12—Ce2^ii^	55.1297 (6)
C2^xviii^—O1—Ce2^vi^	76.818 (2)	O16^ii^—O12—O11	135.4307 (2)
C2^xviii^—O1—O2	92.8170 (1)	O16^ii^—O12—O12^xi^	103.204 (2)
C2^xviii^—O1—O2^vi^	92.8170 (1)	O16^ii^—O12—C6	121.2702 (3)
C2^xviii^—O1—C1	92.881	O4^iii^—O12—O16^ii^	57.7870 (8)
O1—O2—C1	31.9553 (2)	O4^iii^—O12—O17^ii^	61.699 (8)
O2^vi^—O2—O1	59.675 (4)	O4^iii^—O12—Ce1	66.870 (7)
O2^vi^—O2—C1	27.724 (4)	O4^iii^—O12—Ce2^ii^	58.303 (5)
Ce2—O2—O2^vi^	124.181 (5)	O4^iii^—O12—O11	124.264 (4)
Ce2—O2—O1	66.329 (9)	O4^iii^—O12—O12^xi^	158.275 (4)
Ce2—O2—C1	97.265 (9)	O4^iii^—O12—C6	149.808 (4)
Ce3^v^—O2—Ce2	106.246 (8)	O13—O12—O4^iii^	60.189 (2)
Ce3^v^—O2—O2^vi^	123.610 (5)	O13—O12—O16^ii^	86.4014 (16)
Ce3^v^—O2—O1	167.1937 (5)	O13—O12—O17^ii^	99.791 (7)
Ce3^v^—O2—C1	149.7004 (8)	O13—O12—Ce1	52.4738 (19)
O16—O2—Ce3^v^	55.508 (4)	O13—O12—Ce2^ii^	118.036 (7)
O16—O2—Ce2	55.836 (4)	O13—O12—O11	66.837 (6)
O16—O2—O2^vi^	138.329 (5)	O13—O12—O12^xi^	112.055 (4)
O16—O2—O1	113.036 (5)	O13—O12—C6	89.869 (7)
O16—O2—C1	131.621 (6)	O10^ii^—O12—O13	104.0590 (4)
O17^v^—O2—O16	103.383 (5)	O10^ii^—O12—O4^iii^	111.419 (3)
O17^v^—O2—Ce3^v^	53.2950 (5)	O10^ii^—O12—O16^ii^	158.9318 (14)
O17^v^—O2—Ce2	127.563 (6)	O10^ii^—O12—O17^ii^	56.466 (4)
O17^v^—O2—O2^vi^	103.246 (2)	O10^ii^—O12—Ce1	56.451 (2)
O17^v^—O2—O1	139.5006 (1)	O10^ii^—O12—Ce2^ii^	103.9408 (7)
O17^v^—O2—C1	123.6860 (7)	O10^ii^—O12—O11	65.4908 (11)
O8^v^—O2—O17^v^	57.5622 (8)	O10^ii^—O12—O12^xi^	89.9275 (1)
O8^v^—O2—O16	61.433 (8)	O10^ii^—O12—C6	77.6108 (17)
O8^v^—O2—Ce3^v^	55.117 (4)	O7^ii^—O12—O10^ii^	113.4510 (6)
O8^v^—O2—Ce2	71.233 (7)	O7^ii^—O12—O13	142.4895 (9)
O8^v^—O2—O2^vi^	158.555 (3)	O7^ii^—O12—O4^iii^	103.794 (4)
O8^v^—O2—O1	127.228 (3)	O7^ii^—O12—O16^ii^	58.174 (3)
O8^v^—O2—C1	154.027 (4)	O7^ii^—O12—O17^ii^	100.570 (4)
O5^v^—O2—O8^v^	107.485 (4)	O7^ii^—O12—Ce1	157.2170 (6)
O5^v^—O2—O17^v^	66.435 (3)	O7^ii^—O12—Ce2^ii^	53.262 (8)
O5^v^—O2—O16	94.342 (5)	O7^ii^—O12—O11	129.395 (8)
O5^v^—O2—Ce3^v^	55.494 (8)	O7^ii^—O12—O12^xi^	69.418 (3)
O5^v^—O2—Ce2	147.6959 (6)	O7^ii^—O12—C6	98.034 (8)
O5^v^—O2—O2^vi^	68.193 (4)	O8^ii^—O12—O7^ii^	54.5805 (9)
O5^v^—O2—O1	125.173 (8)	O8^ii^—O12—O10^ii^	63.3586 (1)
O5^v^—O2—C1	94.629 (8)	O8^ii^—O12—O13	155.415 (3)
O14^i^—O2—O5^v^	155.7434 (6)	O8^ii^—O12—O4^iii^	103.053 (2)
O14^i^—O2—O8^v^	60.571 (3)	O8^ii^—O12—O16^ii^	99.7859 (1)
O14^i^—O2—O17^v^	90.0405 (19)	O8^ii^—O12—O17^ii^	55.641 (4)
O14^i^—O2—O16	96.969 (7)	O8^ii^—O12—Ce1	105.7797 (10)
O14^i^—O2—Ce3^v^	115.590 (8)	O8^ii^—O12—Ce2^ii^	51.862 (2)
O14^i^—O2—Ce2	52.716 (2)	O8^ii^—O12—O11	119.5629 (15)
O14^i^—O2—O2^vi^	114.450 (4)	O8^ii^—O12—O12^xi^	89.8390 (1)
O14^i^—O2—O1	69.086 (6)	O8^ii^—O12—C6	106.568 (2)
O14^i^—O2—C1	93.773 (7)	Ce1—O13—Ce1^xix^	117.8920 (7)
O14—O2—O14^i^	0.0232 (1)	Ce1^iii^—O13—Ce1	117.8920 (7)
O14—O2—O5^v^	155.7204 (6)	Ce1^iii^—O13—Ce1^xix^	117.8920 (7)
O14—O2—O8^v^	60.564 (3)	O11^xvi^—O13—Ce1^iii^	57.601 (7)
O14—O2—O17^v^	90.0188 (19)	O11^xvi^—O13—Ce1	116.3437 (5)
O14—O2—O16	96.982 (7)	O11^xvi^—O13—Ce1^xix^	115.1258 (3)
O14—O2—Ce3^v^	115.581 (8)	O11^xix^—O13—O11^xvi^	68.973 (8)
O14—O2—Ce2	52.738 (2)	O11^xix^—O13—Ce1^iii^	116.3437 (5)
O14—O2—O2^vi^	114.447 (4)	O11^xix^—O13—Ce1	115.1258 (3)
O14—O2—O1	69.099 (6)	O11^xix^—O13—Ce1^xix^	57.601 (7)
O14—O2—C1	93.779 (7)	O11—O13—O11^xix^	68.973 (8)
O14^v^—O2—O14	0.0181 (1)	O11—O13—O11^xvi^	68.973 (8)
O14^v^—O2—O14^i^	0.0115 (1)	O11—O13—Ce1^iii^	115.1258 (3)
O14^v^—O2—O5^v^	155.7371 (6)	O11—O13—Ce1	57.601 (7)
O14^v^—O2—O8^v^	60.577 (3)	O11—O13—Ce1^xix^	116.3437 (5)
O14^v^—O2—O17^v^	90.0367 (19)	O4—O13—O11	94.279 (8)
O14^v^—O2—O16	96.980 (7)	O4—O13—O11^xix^	100.127 (7)
O14^v^—O2—Ce3^v^	115.595 (8)	O4—O13—O11^xvi^	162.2359 (4)
O14^v^—O2—Ce2	52.726 (2)	O4—O13—Ce1^iii^	139.026 (6)
O14^v^—O2—O2^vi^	114.440 (4)	O4—O13—Ce1	54.3485 (3)
O14^v^—O2—O1	69.083 (6)	O4—O13—Ce1^xix^	66.1074 (4)
O14^v^—O2—C1	93.767 (7)	O4^iii^—O13—O4	94.524 (6)
C3^v^—O2—O14^v^	176.6114 (3)	O4^iii^—O13—O11	100.127 (7)
C3^v^—O2—O14	176.6141 (3)	O4^iii^—O13—O11^xix^	162.2359 (4)
C3^v^—O2—O14^i^	176.6000 (3)	O4^iii^—O13—O11^xvi^	94.279 (8)
C3^v^—O2—O5^v^	26.1478 (6)	O4^iii^—O13—Ce1^iii^	54.3485 (3)
C3^v^—O2—O8^v^	117.446 (3)	O4^iii^—O13—Ce1	66.1074 (4)
C3^v^—O2—O17^v^	90.9548 (17)	O4^iii^—O13—Ce1^xix^	139.026 (6)
C3^v^—O2—O16	79.638 (7)	O4^xix^—O13—O4^iii^	94.524 (6)
C3^v^—O2—Ce3^v^	62.671 (7)	O4^xix^—O13—O4	94.524 (6)
C3^v^—O2—Ce2	124.409 (2)	O4^xix^—O13—O11	162.2359 (4)
C3^v^—O2—O2^vi^	68.457 (3)	O4^xix^—O13—O11^xix^	94.279 (8)
C3^v^—O2—O1	111.958 (6)	O4^xix^—O13—O11^xvi^	100.127 (7)
C3^v^—O2—C1	88.379 (6)	O4^xix^—O13—Ce1^iii^	66.1074 (4)
O6^v^—O2—C3^v^	23.937 (3)	O4^xix^—O13—Ce1	139.026 (6)
O6^v^—O2—O14^v^	152.681 (3)	O4^xix^—O13—Ce1^xix^	54.3485 (3)
O6^v^—O2—O14	152.682 (3)	O12^iii^—O13—O4^xix^	58.248 (7)
O6^v^—O2—O14^i^	152.669 (3)	O12^iii^—O13—O4^iii^	107.398 (3)
O6^v^—O2—O5^v^	43.7297 (15)	O12^iii^—O13—O4	145.4989 (9)
O6^v^—O2—O8^v^	100.9468 (17)	O12^iii^—O13—O11	107.246 (8)
O6^v^—O2—O17^v^	96.2887 (1)	O12^iii^—O13—O11^xix^	64.902 (3)
O6^v^—O2—O16	55.701 (4)	O12^iii^—O13—O11^xvi^	43.5586 (8)
O6^v^—O2—Ce3^v^	51.752 (2)	O12^iii^—O13—Ce1^iii^	53.081 (3)
O6^v^—O2—Ce2	104.0585 (8)	O12^iii^—O13—Ce1	159.6875 (12)
O6^v^—O2—O2^vi^	89.9816 (1)	O12^iii^—O13—Ce1^xix^	80.0898 (14)
O6^v^—O2—O1	118.3820 (14)	O12—O13—O12^iii^	106.768 (4)
O6^v^—O2—C1	104.485 (2)	O12—O13—O4^xix^	145.4989 (9)
O1^v^—O2—O6^v^	147.086 (2)	O12—O13—O4^iii^	58.248 (7)
O1^v^—O2—C3^v^	124.506 (5)	O12—O13—O4	107.398 (3)
O1^v^—O2—O14^v^	58.808 (5)	O12—O13—O11	43.5586 (8)
O1^v^—O2—O14	58.802 (5)	O12—O13—O11^xix^	107.246 (8)
O1^v^—O2—O14^i^	58.819 (5)	O12—O13—O11^xvi^	64.902 (3)
O1^v^—O2—O5^v^	103.564 (3)	O12—O13—Ce1^iii^	80.0898 (14)
O1^v^—O2—O8^v^	92.206 (6)	O12—O13—Ce1	53.081 (3)
O1^v^—O2—O17^v^	65.672 (3)	O12—O13—Ce1^xix^	159.6875 (12)
O1^v^—O2—O16	151.8658 (16)	O12^xix^—O13—O12	106.768 (4)
O1^v^—O2—Ce3^v^	118.936 (2)	O12^xix^—O13—O12^iii^	106.768 (4)
O1^v^—O2—Ce2	108.736 (3)	O12^xix^—O13—O4^xix^	107.398 (3)
O1^v^—O2—O2^vi^	69.422 (3)	O12^xix^—O13—O4^iii^	145.4989 (9)
O1^v^—O2—O1	73.838 (3)	O12^xix^—O13—O4	58.248 (7)
O1^v^—O2—C1	68.961 (3)	O12^xix^—O13—O11	64.902 (3)
O4^iii^—O3—C2	30.207 (4)	O12^xix^—O13—O11^xix^	43.5586 (8)
O4^viii^—O3—O4^iii^	60.392 (9)	O12^xix^—O13—O11^xvi^	107.246 (8)
O4^viii^—O3—C2	30.207 (4)	O12^xix^—O13—Ce1^iii^	159.6875 (12)
Ce1^vi^—O3—O4^viii^	79.987 (8)	O12^xix^—O13—Ce1	80.0898 (14)
Ce1^vi^—O3—O4^iii^	139.9486 (12)	O12^xix^—O13—Ce1^xix^	53.081 (3)
Ce1^vi^—O3—C2	110.107 (3)	Ce2^v^—O14—O14^i^	149.0662 (1)
Ce1—O3—Ce1^vi^	138.078 (7)	Ce2^i^—O14—Ce2^v^	119.5893 (1)
Ce1—O3—O4^viii^	139.9486 (12)	Ce2—O14—Ce2^v^	119.5887 (1)
Ce1—O3—O4^iii^	79.987 (8)	O8^v^—O14—Ce2	70.3681 (2)
Ce1—O3—C2	110.107 (3)	O8^v^—O14—Ce2^i^	145.250 (5)
C2^xix^—O3—Ce1	80.8397 (14)	O8^v^—O14—Ce2^v^	54.3309 (10)
C2^xix^—O3—Ce1^vi^	80.8397 (14)	O8^i^—O14—O8^v^	97.472 (6)
C2^xix^—O3—O4^viii^	126.3958 (19)	O8^i^—O14—Ce2	145.237 (5)
C2^xix^—O3—O4^iii^	126.3958 (19)	O8^i^—O14—Ce2^i^	54.3210 (10)
C2^xix^—O3—C2	132.482	O8^i^—O14—Ce2^v^	70.3783 (2)
O4^vi^—O3—C2^xix^	25.597 (3)	O8^i^—O14—O14^v^	82.3918 (3)
O4^vi^—O3—Ce1	98.412 (6)	O8—O14—O8^i^	97.450 (6)
O4^vi^—O3—Ce1^vi^	55.6339 (17)	O8—O14—O8^v^	97.454 (6)
O4^vi^—O3—O4^viii^	118.180 (7)	O8—O14—Ce2	54.3085 (10)
O4^vi^—O3—O4^iii^	148.7666 (8)	O8—O14—Ce2^i^	70.3504 (2)
O4^vi^—O3—C2	139.4551 (17)	O8—O14—Ce2^v^	145.252 (5)
O4—O3—O4^vi^	44.738 (7)	O8—O14—O14^v^	47.405 (2)
O4—O3—C2^xix^	25.597 (3)	O2^v^—O14—O8	146.7904 (11)
O4—O3—Ce1	55.6339 (17)	O2^v^—O14—O8^i^	58.571 (8)
O4—O3—Ce1^vi^	98.412 (6)	O2^v^—O14—O8^v^	107.572 (3)
O4—O3—O4^viii^	148.7666 (8)	O2^v^—O14—Ce2	155.553 (3)
O4—O3—O4^iii^	118.180 (7)	O2^v^—O14—Ce2^i^	76.6885 (10)
O4—O3—C2	139.4551 (17)	O2^v^—O14—Ce2^v^	53.241 (2)
O17^ix^—O3—O4	136.5832 (14)	O2^v^—O14—O14^v^	102.9520 (4)
O17^ix^—O3—O4^vi^	106.150 (6)	O2—O14—O2^v^	104.086 (4)
O17^ix^—O3—C2^xix^	131.457 (3)	O2—O14—O8	107.538 (3)
O17^ix^—O3—Ce1	120.450 (6)	O2—O14—O8^i^	146.8266 (11)
O17^ix^—O3—Ce1^vi^	53.628 (5)	O2—O14—O8^v^	58.570 (8)
O17^ix^—O3—O4^viii^	66.568 (4)	O2—O14—Ce2	53.230 (2)
O17^ix^—O3—O4^iii^	101.227 (5)	O2—O14—Ce2^i^	155.527 (3)
O17^ix^—O3—C2	83.9598 (4)	O2—O14—Ce2^v^	76.6982 (10)
O17^ii^—O3—O17^ix^	72.154 (10)	O2^i^—O14—O2	104.059 (4)
O17^ii^—O3—O4	106.150 (6)	O2^i^—O14—O2^v^	104.066 (4)
O17^ii^—O3—O4^vi^	136.5832 (14)	O2^i^—O14—O8	58.550 (8)
O17^ii^—O3—C2^xix^	131.457 (3)	O2^i^—O14—O8^i^	107.539 (3)
O17^ii^—O3—Ce1	53.628 (5)	O2^i^—O14—O8^v^	146.7850 (11)
O17^ii^—O3—Ce1^vi^	120.450 (6)	O2^i^—O14—Ce2	76.6673 (10)
O17^ii^—O3—O4^viii^	101.227 (5)	O2^i^—O14—Ce2^i^	53.218 (2)
O17^ii^—O3—O4^iii^	66.568 (4)	O2^i^—O14—Ce2^v^	155.551 (3)
O17^ii^—O3—C2	83.9598 (4)	O2^i^—O14—O14^i^	49.3076 (15)
O6^vi^—O3—O17^ii^	81.261 (5)	O1^v^—O14—O2^i^	103.153 (9)
O6^vi^—O3—O17^ix^	55.6851 (18)	O1^v^—O14—O2	62.863 (3)
O6^vi^—O3—O4	80.954 (3)	O1^v^—O14—O2^v^	42.5738 (11)
O6^vi^—O3—O4^vi^	64.9528 (17)	O1^v^—O14—O8	158.1834 (5)
O6^vi^—O3—C2^xix^	83.6314 (1)	O1^v^—O14—O8^i^	99.772 (7)
O6^vi^—O3—Ce1	90.621 (7)	O1^v^—O14—O8^v^	93.491 (7)
O6^vi^—O3—Ce1^vi^	50.0433 (2)	O1^v^—O14—Ce2	113.0616 (15)
O6^vi^—O3—O4^viii^	118.333 (6)	O1^v^—O14—Ce2^i^	109.7061 (11)
O6^vi^—O3—O4^iii^	145.7234 (8)	O1^v^—O14—Ce2^v^	54.845 (6)
O6^vi^—O3—C2	139.5517 (15)	O1^v^—O14—O14^i^	123.783 (4)
O6—O3—O6^vi^	40.933 (7)	O1—O14—O1^v^	66.021 (8)
O6—O3—O17^ii^	55.6851 (18)	O1—O14—O2^i^	62.846 (3)
O6—O3—O17^ix^	81.261 (5)	O1—O14—O2	42.5667 (11)
O6—O3—O4	64.9528 (17)	O1—O14—O2^v^	103.163 (9)
O6—O3—O4^vi^	80.954 (3)	O1—O14—O8	93.457 (7)
O6—O3—C2^xix^	83.6314 (1)	O1—O14—O8^i^	158.2012 (5)
O6—O3—Ce1	50.0433 (2)	O1—O14—O8^v^	99.761 (7)
O6—O3—Ce1^vi^	90.621 (7)	O1—O14—Ce2	54.827 (6)
O6—O3—O4^viii^	145.7234 (8)	O1—O14—Ce2^i^	113.0400 (15)
O6—O3—O4^iii^	118.333 (6)	O1—O14—Ce2^v^	109.7157 (11)
O6—O3—C2	139.5517 (15)	O1^x^—O14—O1	66.012 (8)
O3^xix^—O4—C2^xix^	30.7598 (1)	O1^x^—O14—O1^v^	66.021 (8)
O4^vi^—O4—O3^xix^	59.804 (4)	O1^x^—O14—O2^i^	42.5606 (11)
O4^vi^—O4—C2^xix^	29.068 (4)	O1^x^—O14—O2	103.155 (9)
Ce1—O4—O4^vi^	118.636 (4)	O1^x^—O14—O2^v^	62.858 (3)
Ce1—O4—O3^xix^	121.577 (3)	O1^x^—O14—O8	99.738 (7)
Ce1—O4—C2^xix^	124.405 (3)	O1^x^—O14—O8^i^	93.473 (7)
Ce2^i^—O4—Ce1	107.315 (7)	O1^x^—O14—O8^v^	158.2146 (5)
Ce2^i^—O4—O4^vi^	121.820 (5)	O1^x^—O14—Ce2	109.6839 (11)
Ce2^i^—O4—O3^xix^	121.554 (3)	O1^x^—O14—Ce2^i^	54.827 (6)
Ce2^i^—O4—C2^xix^	128.183 (4)	O1^x^—O14—Ce2^v^	113.0717 (15)
O16^i^—O4—Ce2^i^	54.116 (4)	Ce3^ii^—O15—O15^iv^	92.5393 (1)
O16^i^—O4—Ce1	54.511 (3)	Ce3^iv^—O15—Ce3^ii^	119.2634 (3)
O16^i^—O4—O4^vi^	138.003 (5)	Ce3—O15—Ce3^iv^	119.2199 (3)
O16^i^—O4—O3^xix^	162.1872 (6)	Ce3—O15—Ce3^ii^	119.2221 (3)
O16^i^—O4—C2^xix^	166.9973 (8)	O5^ii^—O15—Ce3	112.6239 (11)
O3—O4—O16^i^	103.575 (4)	O5^ii^—O15—Ce3^iv^	112.0623 (10)
O3—O4—Ce2^i^	156.3470 (4)	O5^ii^—O15—Ce3^ii^	56.343 (6)
O3—O4—Ce1	53.878 (7)	O5^ii^—O15—O15^ii^	123.878 (4)
O3—O4—O4^vi^	67.631 (4)	O5^xviii^—O15—O5^ii^	65.593 (8)
O3—O4—O3^xix^	82.098 (3)	O5^xviii^—O15—Ce3	112.0294 (10)
O3—O4—C2^xix^	71.541 (4)	O5^xviii^—O15—Ce3^iv^	56.342 (6)
O17^i^—O4—O3	150.7229 (1)	O5^xviii^—O15—Ce3^ii^	112.6562 (11)
O17^i^—O4—O16^i^	101.251 (5)	O5—O15—O5^xviii^	65.585 (8)
O17^i^—O4—Ce2^i^	52.4576 (5)	O5—O15—O5^ii^	65.585 (8)
O17^i^—O4—Ce1	137.099 (6)	O5—O15—Ce3	56.324 (6)
O17^i^—O4—O4^vi^	102.892 (2)	O5—O15—Ce3^iv^	112.6328 (11)
O17^i^—O4—O3^xix^	69.711 (4)	O5—O15—Ce3^ii^	112.0412 (10)
O17^i^—O4—C2^xix^	87.123 (4)	O5—O15—O15^iv^	56.102 (4)
O12^xix^—O4—O17^i^	56.1243 (12)	O10^iv^—O15—O5	157.9793 (4)
O12^xix^—O4—O3	127.1113 (13)	O10^iv^—O15—O5^xviii^	93.171 (8)
O12^xix^—O4—O16^i^	59.500 (8)	O10^iv^—O15—O5^ii^	101.324 (6)
O12^xix^—O4—Ce2^i^	52.425 (4)	O10^iv^—O15—Ce3	143.657 (5)
O12^xix^—O4—Ce1	81.312 (7)	O10^iv^—O15—Ce3^iv^	53.9975 (8)
O12^xix^—O4—O4^vi^	158.275 (4)	O10^iv^—O15—Ce3^ii^	69.5063 (1)
O12^xix^—O4—O3^xix^	103.490 (7)	O10^ii^—O15—O10^iv^	96.910 (6)
O12^xix^—O4—C2^xix^	133.190 (7)	O10^ii^—O15—O5	101.288 (6)
O13—O4—O12^xix^	61.563 (5)	O10^ii^—O15—O5^xviii^	157.9644 (4)
O13—O4—O17^i^	97.026 (3)	O10^ii^—O15—O5^ii^	93.155 (8)
O13—O4—O3	68.714 (6)	O10^ii^—O15—Ce3	69.4791 (1)
O13—O4—O16^i^	88.112 (8)	O10^ii^—O15—Ce3^iv^	143.671 (5)
O13—O4—Ce2^i^	113.573 (9)	O10^ii^—O15—Ce3^ii^	53.9861 (8)
O13—O4—Ce1	52.846 (3)	O10—O15—O10^ii^	96.888 (6)
O13—O4—O4^vi^	121.996 (5)	O10—O15—O10^iv^	96.906 (6)
O13—O4—O3^xix^	78.146 (7)	O10—O15—O5	93.137 (8)
O13—O4—C2^xix^	100.832 (8)	O10—O15—O5^xviii^	101.299 (6)
Ce1^xix^—O4—O13	49.0432 (19)	O10—O15—O5^ii^	157.9469 (4)
Ce1^xix^—O4—O12^xix^	49.9723 (12)	O10—O15—Ce3	53.9753 (8)
Ce1^xix^—O4—O17^i^	49.9141 (6)	O10—O15—Ce3^iv^	69.4877 (1)
Ce1^xix^—O4—O3	106.836 (2)	O10—O15—Ce3^ii^	143.661 (5)
Ce1^xix^—O4—O16^i^	108.066 (9)	O10—O15—O15^ii^	34.844 (4)
Ce1^xix^—O4—Ce2^i^	89.482 (4)	O10—O15—O15^iv^	79.8674 (5)
Ce1^xix^—O4—Ce1	100.042 (5)	O6^ii^—O15—O10	146.6261 (11)
Ce1^xix^—O4—O4^vi^	113.802 (4)	O6^ii^—O15—O10^ii^	107.530 (3)
Ce1^xix^—O4—O3^xix^	54.244 (8)	O6^ii^—O15—O10^iv^	58.523 (8)
Ce1^xix^—O4—C2^xix^	84.936 (8)	O6^ii^—O15—O5	103.685 (9)
O1^x^—O4—Ce1^xix^	115.4973 (19)	O6^ii^—O15—O5^xviii^	61.872 (3)
O1^x^—O4—O13	162.7380 (3)	O6^ii^—O15—O5^ii^	44.2501 (12)
O1^x^—O4—O12^xix^	103.338 (5)	O6^ii^—O15—Ce3	156.856 (2)
O1^x^—O4—O17^i^	66.285 (3)	O6^ii^—O15—Ce3^iv^	77.4263 (11)
O1^x^—O4—O3	128.095 (6)	O6^ii^—O15—Ce3^ii^	53.548 (2)
O1^x^—O4—O16^i^	90.856 (5)	O6^ii^—O15—O15^ii^	149.900 (3)
O1^x^—O4—Ce2^i^	53.331 (8)	O6^iv^—O15—O6^ii^	104.618 (4)
O1^x^—O4—Ce1	137.3266 (12)	O6^iv^—O15—O10	58.503 (8)
O1^x^—O4—O4^vi^	68.644 (3)	O6^iv^—O15—O10^ii^	146.5854 (11)
O1^x^—O4—O3^xix^	98.899 (4)	O6^iv^—O15—O10^iv^	107.528 (3)
O1^x^—O4—C2^xix^	83.374 (5)	O6^iv^—O15—O5	61.855 (3)
C1^x^—O4—O1^x^	24.2478 (6)	O6^iv^—O15—O5^xviii^	44.2430 (12)
C1^x^—O4—Ce1^xix^	138.7154 (11)	O6^iv^—O15—O5^ii^	103.681 (9)
C1^x^—O4—O13	165.0058 (7)	O6^iv^—O15—Ce3	77.3946 (11)
C1^x^—O4—O12^xix^	111.816 (3)	O6^iv^—O15—Ce3^iv^	53.535 (2)
C1^x^—O4—O17^i^	88.8098 (16)	O6^iv^—O15—Ce3^ii^	156.880 (2)
C1^x^—O4—O3	111.607 (4)	O6^iv^—O15—O15^ii^	46.5982 (16)
C1^x^—O4—O16^i^	77.183 (7)	O6—O15—O6^iv^	104.591 (4)
C1^x^—O4—Ce2^i^	59.753 (7)	O6—O15—O6^ii^	104.612 (4)
C1^x^—O4—Ce1	114.369 (2)	O6—O15—O10	107.496 (3)
C1^x^—O4—O4^vi^	69.543 (3)	O6—O15—O10^ii^	58.503 (8)
C1^x^—O4—O3^xix^	116.848 (6)	O6—O15—O10^iv^	146.6225 (11)
C1^x^—O4—C2^xix^	93.222 (7)	O6—O15—O5	44.2362 (12)
O6—O5—C3	29.324 (4)	O6—O15—O5^xviii^	103.675 (9)
O6^vi^—O5—O6	58.527 (9)	O6—O15—O5^ii^	61.860 (3)
O6^vi^—O5—C3	29.324 (4)	O6—O15—Ce3	53.525 (2)
Ce3^vi^—O5—O6^vi^	61.702 (5)	O6—O15—Ce3^iv^	156.852 (2)
Ce3^vi^—O5—O6	118.490 (4)	O6—O15—Ce3^ii^	77.4061 (11)
Ce3^vi^—O5—C3	89.6415 (1)	O6—O15—O15^ii^	79.7232 (3)
Ce3—O5—Ce3^vi^	153.211 (5)	Ce2—O16—Ce1^v^	116.455 (3)
Ce3—O5—O6^vi^	118.490 (4)	Ce3^v^—O16—Ce2	109.778 (2)
Ce3—O5—O6	61.702 (5)	Ce3^v^—O16—Ce1^v^	109.321 (3)
Ce3—O5—C3	89.6415 (1)	O6^v^—O16—Ce3^v^	56.9826 (4)
C3^xviii^—O5—Ce3	97.2823 (12)	O6^v^—O16—Ce2	115.020 (8)
C3^xviii^—O5—Ce3^vi^	97.2823 (12)	O6^v^—O16—Ce1^v^	56.1509 (17)
C3^xviii^—O5—O6^vi^	136.609 (3)	O2—O16—O6^v^	67.774 (8)
C3^xviii^—O5—O6	136.609 (3)	O2—O16—Ce3^v^	58.7371 (6)
C3^xviii^—O5—C3	148.374	O2—O16—Ce2	56.5030 (7)
O2^i^—O5—C3^xviii^	130.3207 (12)	O2—O16—Ce1^v^	111.212 (8)
O2^i^—O5—Ce3	55.0755 (13)	O4^v^—O16—O2	70.397 (8)
O2^i^—O5—Ce3^vi^	98.642 (6)	O4^v^—O16—O6^v^	72.106 (8)
O2^i^—O5—O6^vi^	91.877 (4)	O4^v^—O16—Ce3^v^	117.374 (8)
O2^i^—O5—O6	70.706 (3)	O4^v^—O16—Ce2	60.3963 (11)
O2^i^—O5—C3	78.3561 (3)	O4^v^—O16—Ce1^v^	57.6204 (19)
O2^x^—O5—O2^i^	43.614 (7)	O12^iv^—O16—O4^v^	62.713 (7)
O2^x^—O5—C3^xviii^	130.3207 (12)	O12^iv^—O16—O2	109.2648 (15)
O2^x^—O5—Ce3	98.642 (6)	O12^iv^—O16—O6^v^	131.7652 (10)
O2^x^—O5—Ce3^vi^	55.0755 (13)	O12^iv^—O16—Ce3^v^	163.7652 (1)
O2^x^—O5—O6^vi^	70.706 (3)	O12^iv^—O16—Ce2	55.099 (2)
O2^x^—O5—O6	91.877 (4)	O12^iv^—O16—Ce1^v^	84.7280 (18)
O2^x^—O5—C3	78.3561 (3)	O10^xxi^—O16—O12^iv^	124.442 (2)
O15^xviii^—O5—O2^x^	106.476 (8)	O10^xxi^—O16—O4^v^	116.5344 (8)
O15^xviii^—O5—O2^i^	149.6999 (13)	O10^xxi^—O16—O2	122.8147 (1)
O15^xviii^—O5—C3^xviii^	68.856 (2)	O10^xxi^—O16—O6^v^	62.712 (7)
O15^xviii^—O5—Ce3	154.676 (3)	O10^xxi^—O16—Ce3^v^	71.0059 (16)
O15^xviii^—O5—Ce3^vi^	52.094 (7)	O10^xxi^—O16—Ce2	176.9096 (3)
O15^xviii^—O5—O6^vi^	68.538 (5)	O10^xxi^—O16—Ce1^v^	60.660 (3)
O15^xviii^—O5—O6	113.393 (5)	O8^v^—O16—O10^xxi^	110.8188 (15)
O15^xviii^—O5—C3	92.2522 (2)	O8^v^—O16—O12^iv^	109.5526 (15)
O15^iv^—O5—O15^xviii^	102.588 (10)	O8^v^—O16—O4^v^	125.5057 (2)
O15^iv^—O5—O2^x^	149.6999 (13)	O8^v^—O16—O2	61.956 (7)
O15^iv^—O5—O2^i^	106.476 (8)	O8^v^—O16—O6^v^	109.8427 (14)
O15^iv^—O5—C3^xviii^	68.856 (2)	O8^v^—O16—Ce3^v^	56.096 (2)
O15^iv^—O5—Ce3	52.094 (7)	O8^v^—O16—Ce2	71.7400 (17)
O15^iv^—O5—Ce3^vi^	154.676 (3)	O8^v^—O16—Ce1^v^	165.3829 (4)
O15^iv^—O5—O6^vi^	113.393 (5)	O9^v^—O16—O8^v^	65.9361 (11)
O15^iv^—O5—O6	68.538 (5)	O9^v^—O16—O10^xxi^	47.2690 (1)
O15^iv^—O5—C3	92.2522 (2)	O9^v^—O16—O12^iv^	129.743 (7)
O15^ix^—O5—O15^iv^	102.587 (10)	O9^v^—O16—O4^v^	162.2969 (8)
O15^ix^—O5—O15^xviii^	0.0240 (1)	O9^v^—O16—O2	110.502 (6)
O15^ix^—O5—O2^x^	106.481 (8)	O9^v^—O16—O6^v^	91.626 (9)
O15^ix^—O5—O2^i^	149.7077 (13)	O9^v^—O16—Ce3^v^	54.959 (7)
O15^ix^—O5—C3^xviii^	68.834 (2)	O9^v^—O16—Ce2	135.7052 (2)
O15^ix^—O5—Ce3	154.675 (3)	O9^v^—O16—Ce1^v^	107.723 (3)
O15^ix^—O5—Ce3^vi^	52.095 (7)	O7—O16—O9^v^	83.807 (7)
O15^ix^—O5—O6^vi^	68.561 (5)	O7—O16—O8^v^	45.7675 (2)
O15^ix^—O5—O6	113.416 (5)	O7—O16—O10^xxi^	126.283 (7)
O15^ix^—O5—C3	92.2762 (2)	O7—O16—O12^iv^	66.3854 (12)
O15^ii^—O5—O15^ix^	102.586 (10)	O7—O16—O4^v^	113.882 (6)
O15^ii^—O5—O15^iv^	0.0240 (1)	O7—O16—O2	90.455 (9)
O15^ii^—O5—O15^xviii^	102.587 (10)	O7—O16—O6^v^	154.6495 (16)
O15^ii^—O5—O2^x^	149.7077 (13)	O7—O16—Ce3^v^	101.157 (2)
O15^ii^—O5—O2^i^	106.481 (8)	O7—O16—Ce2	56.702 (7)
O15^ii^—O5—C3^xviii^	68.834 (2)	O7—O16—Ce1^v^	148.6845 (1)
O15^ii^—O5—Ce3	52.095 (7)	C5^xxii^—O16—O7	100.979 (9)
O15^ii^—O5—Ce3^vi^	154.675 (3)	C5^xxii^—O16—O9^v^	26.0922 (16)
O15^ii^—O5—O6^vi^	113.416 (5)	C5^xxii^—O16—O8^v^	91.942 (3)
O15^ii^—O5—O6	68.561 (5)	C5^xxii^—O16—O10^xxi^	25.396 (2)
O15^ii^—O5—C3	92.2762 (2)	C5^xxii^—O16—O12^iv^	120.634 (5)
O15^vi^—O5—O15^ii^	102.570 (10)	C5^xxii^—O16—O4^v^	140.500 (3)
O15^vi^—O5—O15^ix^	0.0204 (1)	C5^xxii^—O16—O2	129.315 (4)
O15^vi^—O5—O15^iv^	102.571 (10)	C5^xxii^—O16—O6^v^	84.474 (10)
O15^vi^—O5—O15^xviii^	0.0199 (1)	C5^xxii^—O16—Ce3^v^	70.598 (5)
O15^vi^—O5—O2^x^	106.494 (8)	C5^xxii^—O16—Ce2	157.6434 (18)
O15^vi^—O5—O2^i^	149.7190 (13)	C5^xxii^—O16—Ce1^v^	82.962 (5)
O15^vi^—O5—C3^xviii^	68.838 (2)	C4^v^—O16—C5^xxii^	87.277 (6)
O15^vi^—O5—Ce3	154.659 (3)	C4^v^—O16—O7	24.8799 (15)
O15^vi^—O5—Ce3^vi^	52.110 (7)	C4^v^—O16—O9^v^	64.702 (4)
O15^vi^—O5—O6^vi^	68.554 (5)	C4^v^—O16—O8^v^	24.782 (2)
O15^vi^—O5—O6	113.398 (5)	C4^v^—O16—O10^xxi^	111.597 (4)
O15^vi^—O5—C3	92.2639 (2)	C4^v^—O16—O12^iv^	91.226 (3)
O15—O5—O15^vi^	102.555 (10)	C4^v^—O16—O4^v^	131.854 (4)
O15—O5—O15^ii^	0.0204 (1)	C4^v^—O16—O2	83.166 (10)
O15—O5—O15^ix^	102.570 (10)	C4^v^—O16—O6^v^	133.416 (3)
O15—O5—O15^iv^	0.0199 (1)	C4^v^—O16—Ce3^v^	77.038 (4)
O15—O5—O15^xviii^	102.571 (10)	C4^v^—O16—Ce2	71.465 (5)
O15—O5—O2^x^	149.7190 (13)	C4^v^—O16—Ce1^v^	165.6060 (15)
O15—O5—O2^i^	106.494 (8)	O11^v^—O16—C4^v^	110.266 (9)
O15—O5—C3^xviii^	68.838 (2)	O11^v^—O16—C5^xxii^	60.155 (4)
O15—O5—Ce3	52.110 (7)	O11^v^—O16—O7	99.222 (8)
O15—O5—Ce3^vi^	154.659 (3)	O11^v^—O16—O9^v^	82.088 (6)
O15—O5—O6^vi^	113.398 (5)	O11^v^—O16—O8^v^	132.633 (7)
O15—O5—O6	68.554 (5)	O11^v^—O16—O10^xxi^	59.5288 (8)
O15—O5—C3	92.2639 (2)	O11^v^—O16—O12^iv^	65.1525 (11)
O6^xviii^—O5—O15	92.961 (5)	O11^v^—O16—O4^v^	95.108 (8)
O6^xviii^—O5—O15^vi^	59.525 (2)	O11^v^—O16—O2	165.0132 (2)
O6^xviii^—O5—O15^ii^	92.958 (5)	O11^v^—O16—O6^v^	104.840 (7)
O6^xviii^—O5—O15^ix^	59.513 (2)	O11^v^—O16—Ce3^v^	129.3030 (6)
O6^xviii^—O5—O15^iv^	92.979 (5)	O11^v^—O16—Ce2	120.2245 (13)
O6^xviii^—O5—O15^xviii^	59.537 (2)	O11^v^—O16—Ce1^v^	55.561 (8)
O6^xviii^—O5—O2^x^	108.957 (4)	Ce2—O17—Ce3	114.113 (3)
O6^xviii^—O5—O2^i^	126.2239 (14)	Ce1^iv^—O17—Ce2	107.1224 (19)
O6^xviii^—O5—C3^xviii^	24.181 (3)	Ce1^iv^—O17—Ce3	115.234 (2)
O6^xviii^—O5—Ce3	115.181 (3)	O12^iv^—O17—Ce1^iv^	56.9200 (1)
O6^xviii^—O5—Ce3^vi^	74.258 (4)	O12^iv^—O17—Ce2	56.4163 (11)
O6^xviii^—O5—O6^vi^	125.720 (7)	O12^iv^—O17—Ce3	112.336 (8)
O6^xviii^—O5—O6	159.0602 (2)	O8—O17—O12^iv^	69.705 (8)
O6^xviii^—O5—C3	151.767 (2)	O8—O17—Ce1^iv^	118.309 (7)
O6^iv^—O5—O6^xviii^	41.972 (7)	O8—O17—Ce2	57.4639 (13)
O6^iv^—O5—O15	59.525 (2)	O8—O17—Ce3	58.6014 (16)
O6^iv^—O5—O15^vi^	92.961 (5)	O10—O17—O8	72.509 (8)
O6^iv^—O5—O15^ii^	59.513 (2)	O10—O17—O12^iv^	68.464 (8)
O6^iv^—O5—O15^ix^	92.958 (5)	O10—O17—Ce1^iv^	61.3787 (6)
O6^iv^—O5—O15^iv^	59.537 (2)	O10—O17—Ce2	114.192 (8)
O6^iv^—O5—O15^xviii^	92.979 (5)	O10—O17—Ce3	57.0140 (15)
O6^iv^—O5—O2^x^	126.2239 (14)	O2^i^—O17—O10	113.5901 (11)
O6^iv^—O5—O2^i^	108.957 (4)	O2^i^—O17—O8	62.846 (7)
O6^iv^—O5—C3^xviii^	24.181 (3)	O2^i^—O17—O12^iv^	127.9074 (5)
O6^iv^—O5—Ce3	74.258 (4)	O2^i^—O17—Ce1^iv^	172.4223 (1)
O6^iv^—O5—Ce3^vi^	115.181 (3)	O2^i^—O17—Ce2	79.9470 (17)
O6^iv^—O5—O6^vi^	159.0602 (2)	O2^i^—O17—Ce3	58.298 (3)
O6^iv^—O5—O6	125.720 (7)	O6^iv^—O17—O2^i^	119.0939 (18)
O6^iv^—O5—C3	151.767 (2)	O6^iv^—O17—O10	62.300 (7)
O6^vi^—O6—C3	29.271 (4)	O6^iv^—O17—O8	130.9954 (8)
O5—O6—O6^vi^	60.737 (4)	O6^iv^—O17—O12^iv^	107.5822 (17)
O5—O6—C3	31.5978 (1)	O6^iv^—O17—Ce1^iv^	53.992 (2)
Ce1—O6—O5	167.1642 (6)	O6^iv^—O17—Ce2	160.8261 (1)
Ce1—O6—O6^vi^	119.721 (4)	O6^iv^—O17—Ce3	80.6192 (18)
Ce1—O6—C3	146.4943 (5)	O4^v^—O17—O6^iv^	105.9960 (14)
Ce3—O6—Ce1	108.727 (8)	O4^v^—O17—O2^i^	119.8010 (18)
Ce3—O6—O5	66.187 (9)	O4^v^—O17—O10	122.1115 (1)
Ce3—O6—O6^vi^	124.934 (5)	O4^v^—O17—O8	113.6528 (10)
Ce3—O6—C3	96.234 (9)	O4^v^—O17—O12^iv^	62.176 (7)
O16^i^—O6—Ce3	56.702 (5)	O4^v^—O17—Ce1^iv^	67.1743 (16)
O16^i^—O6—Ce1	55.793 (4)	O4^v^—O17—Ce2	58.546 (3)
O16^i^—O6—O5	114.457 (5)	O4^v^—O17—Ce3	172.2465 (6)
O16^i^—O6—O6^vi^	138.988 (5)	O3^xviii^—O17—O4^v^	43.7209 (4)
O16^i^—O6—C3	131.257 (6)	O3^xviii^—O17—O6^iv^	65.2125 (11)
O17^ii^—O6—O16^i^	102.930 (5)	O3^xviii^—O17—O2^i^	129.089 (7)
O17^ii^—O6—Ce3	126.105 (6)	O3^xviii^—O17—O10	111.597 (6)
O17^ii^—O6—Ce1	55.4436 (7)	O3^xviii^—O17—O8	156.4651 (13)
O17^ii^—O6—O5	137.3094 (1)	O3^xviii^—O17—O12^iv^	89.855 (9)
O17^ii^—O6—O6^vi^	103.146 (2)	O3^xviii^—O17—Ce1^iv^	53.068 (6)
O17^ii^—O6—C3	124.8427 (8)	O3^xviii^—O17—Ce2	101.844 (3)
O10^ii^—O6—O17^ii^	57.6169 (8)	O3^xviii^—O17—Ce3	143.8805 (3)
O10^ii^—O6—O16^i^	60.828 (8)	C2^xviii^—O17—O3^xviii^	24.1798 (14)
O10^ii^—O6—Ce3	70.020 (7)	C2^xviii^—O17—O4^v^	23.871 (2)
O10^ii^—O6—Ce1	59.763 (4)	C2^xviii^—O17—O6^iv^	89.365 (3)
O10^ii^—O6—O5	125.212 (3)	C2^xviii^—O17—O2^i^	117.383 (5)
O10^ii^—O6—O6^vi^	158.488 (3)	C2^xviii^—O17—O10	128.893 (4)
O10^ii^—O6—C3	153.137 (4)	C2^xviii^—O17—O8	136.011 (3)
O15^iv^—O6—O10^ii^	60.372 (3)	C2^xviii^—O17—O12^iv^	83.146 (10)
O15^iv^—O6—O17^ii^	88.7766 (18)	C2^xviii^—O17—Ce1^iv^	67.539 (4)
O15^iv^—O6—O16^i^	97.647 (7)	C2^xviii^—O17—Ce2	78.793 (4)
O15^iv^—O6—Ce3	52.394 (2)	C2^xviii^—O17—Ce3	163.4768 (15)

Symmetry codes: (i) −*y*+1, *x*−*y*, *z*; (ii) −*x*+*y*, −*x*+1, *z*; (iii) −*y*, *x*−*y*, *z*; (iv) −*y*+1, *x*−*y*+1, *z*; (v) −*x*+*y*+1, −*x*+1, *z*; (vi) *x*, *y*, −*z*; (vii) −*x*+*y*+1, −*x*+1, −*z*; (viii) −*y*, *x*−*y*, −*z*; (ix) −*x*+*y*, −*x*+1, −*z*; (x) −*y*+1, *x*−*y*, −*z*; (xi) *x*, *y*, −*z*+1; (xii) −*y*+1, *x*−*y*, −*z*+1; (xiii) −*y*+1, *x*−*y*+1, −*z*+1; (xiv) −*x*+*y*+1, −*x*+2, *z*; (xv) −*x*+*y*+1, −*x*+2, −*z*+1; (xvi) −*y*, *x*−*y*, −*z*+1; (xvii) −*x*+*y*, −*x*+1, −*z*+1; (xviii) −*y*+1, *x*−*y*+1, −*z*; (xix) −*x*+*y*, −*x*, *z*; (xx) −*x*+*y*+1, −*x*+1, −*z*+1; (xxi) −*y*+2, *x*−*y*+1, *z*; (xxii) −*y*+2, *x*−*y*+1, −*z*+1.
